# Active Oscillations of Elastic Membranes Arising from the Coupling with a Catalytic Reaction

**DOI:** 10.1002/advs.77877

**Published:** 2026-10-08

**Authors:** Alberto Comoretto, Samuel D. Gollob, Erik Steur, Kaitlyn P. Becker, Ellen T. Roche, Johannes T.B. Overvelde

**Affiliations:** ^1^ Autonomous Matter Department AMOLF Amsterdam The Netherlands; ^2^ Mechanical Engineering Dept. Massachusetts Institute of Technology (MIT) Cambridge USA; ^3^ Institute for Complex Molecular Systems Department of Mechanical Engineering Eindhoven University of Technology Eindhoven The Netherlands; ^4^ Institute for Medical Engineering and Science Massachusetts Institute of Technology (MIT) Cambridge USA

**Keywords:** chemical power, embodied energy, physical control, self‐oscillations, soft robotics

## Abstract

Soft fluidic machines increasingly rely on self‐sustained oscillations to accomplish tasks autonomously without electronics or software. Typically, these oscillations arise from mechanical nonlinearities in complex geometries or relatively involved fluidic circuitry, and are passive in that they require external power sources. Here, we show that, by harnessing the coupling with a catalytic reaction, active oscillations can arise even in flat elastic membranes without fluidic circuits or external power. We embed a silver catalyst in a flat elastic membrane suspended over a pool of liquid hydrogen peroxide as fuel. When the fuel contacts the catalyst, it decomposes, releasing gases and generating pressure. We observe an oscillation where the reaction activates and deactivates spontaneously, causing the membrane to cyclically inflate and deflate for hours consecutively. Using experiments and a lumped‐parameter model, we show that the oscillation arises from an interplay between catalyst capillarity, membrane stiffness, and venting resistance, which together induce negative feedback and separation of timescales. These results demonstrate an active oscillation in which the energy release is directly coupled to the oscillation dynamics. By embodying energy within the oscillation, we expand the library of phenomena to achieve self‐oscillation in autonomous soft machines, opening avenues for elasto‐chemical strategies for physical control.

## Graphical abstract

Coupling an elastic membrane to a catalytic reaction produces active oscillations that self‐sustain for hours: the membrane cyclically inflates and deflates as the reaction spontaneously activates and deactivates, without external power. Energy release is directly embodied in the oscillation dynamics, opening elasto‐chemical strategies for the physical control of soft machines.

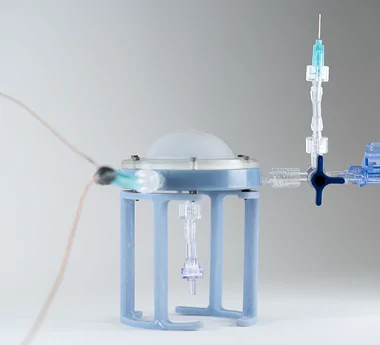

## 1 Introduction

Soft machines [1, 2] hold great promise for autonomous operation in real‐world scenarios [3]. The intrinsic compliance of their bodies leads to features that distinguish them from rigid machines, such as passive adaptability [4, 5, 6], exhibition of complex output deformations from simple inputs [7], and resilience to damage [8, 9].

Relatively recently, fluidic circuits [10] have been identified as a promising platform to increasingly move autonomy capabilities from a central electronic processor to the machine's soft body itself, a practice denoted as physical control [11]. Fluidic circuits, consisting of networks of centimeter‐scale tubings [12, 13, 14] and nonlinear inflatable elements [15, 16], allow for internal sequencing of complex motions given simple pneumatic inputs. For example, given a cyclic pressure drive, completely fluidic robots sequence leg movements to locomote [17], and even play Beethoven's Ninth Symphony [18].

To achieve such functionalities, these systems [17, 18] require an externally timed pressure signal. To increase the independence from external computers, fluidic circuits can generate their own timing signals internally, harnessing self‐oscillation [19]. Given a constant input, self‐oscillating fluidic circuits [20, 21, 22, 23, 24, 25, 26, 27] embedded in the machines' bodies generate oscillatory pressure signals, sequencing the actions of the machines. For example, fluidic relaxation oscillators obtain cyclic activation given a constant inflow of air through a single hysteretic valve [23, 26]; the valve alternates between a closed and an open state, snapping between each state due to its nonlinear characteristic curve. Fluidic ring oscillators [28], given constant input pressure, display a pressure wave that travels along a ring of three interconnected valves, sequentially inflating limbs of a locomoting robot [29]. In our previous work, self‐oscillating limbs made of kinked elastic tubes display cyclic, asymmetric motions when connected to an external mini air pump, resulting in responsive, synchronized gaits across various environments [20].

In general, fluidic circuits rely on mechanical nonlinearities to self‐oscillate, where hysteretic behavior arises from microfluidic networks [30, 31] or macroscopic elements with specific geometric designs. For instance, the relaxation oscillator [23, 26] relies on a shell with a non‐monotonic pressure‐volume curve and with a cut at its pole [32]; the ring oscillator [28] requires multiple bistable valves, each assembled from a bistable shell, an external soft capsule, and two kinking tubes [21]; the self‐oscillating limb leverages a thin elastic tube along which kink instabilities spontaneously form, travel, and disappear [20].

These self‐oscillations are passive, as they rely on an external pneumatic power supply, often in the form of a compressor. Even when untethered, the power supply is a separate component placed onboard as relatively bulky pumps and batteries [20, 22] or pressure canisters [25, 29]. To remove rigid parts associated with the power supply, researchers have harnessed liquid fuels that rapidly decompose into gases when in contact with a catalyst [33, 34], directly inflating soft limbs [30]. However, even in this scenario, the energy system is external to the passive self‐oscillating system, which still requires a relatively complex network of gates and microfluidic components [31] to generate cyclic activation.

Outside the realm of fluidics, self‐shadowing effects are commonly used to obtain oscillations in carefully designed materials. For instance, strips of photoactive polymers undergo self‐oscillations when illuminated with constant light [35, 36, 37, 38]. In chemo‐mechano‐chemical homeostatic materials, catalytic microstructures reversibly move into and out of a reactant layer when their supporting hydrogel swells and deswells, thereby regulating chemical reactions that, in turn, feed back on the hydrogel swelling state, closing a self‐regulating loop [39]. Self‐oscillating polymer brushes as artificial cilia use Belousov–Zhabotinsky redox waves to drive autonomous nanoscale swelling‐deswelling cycles with controlled unidirectional propagation [40]. In general, in these systems, the stimulus‐induced activation leads to shape changes that cause subsequent suppression of the stimulus, effectively resetting the system for the next cycle. Crucially, in these systems, the access to the energy source is directly dependent on the internal states of the oscillating dynamics. In other words, the energy‐consuming feature typical of active matter [41, 42, 43, 44]—for which energy gets dissipated internally—enters directly as a state variable of the oscillators. Therefore, here we refer to such systems as active oscillators.

Such coupling between energy access and internal states allows mechanisms with relatively simple responses (for instance monotonically bending beams [45, 46]) to undergo out‐of‐equilibrium oscillations. This active oscillation architecture contrasts with passive fluidic self‐oscillators, which instead require elements with highly nonlinear mechanical responses, such as mechanical instabilities [20, 22, 23, 25, 29, 30]. We hypothesize that this reliance on nonlinear mechanical elements arises because the passive oscillating subsystem is decoupled from the subsystem providing the energy input. On the other hand, compared with active self‐oscillating materials, passive self‐oscillating fluidic circuits have a distinct feature: their output is an oscillatory pressure or flow signal that can directly drive the actuation and sequencing of soft devices to perform specific functions [11], such as locomotion [20], manipulation [47], and self‐sensing [48].

Here, we aim to bridge these two approaches and combine the advantages from both. We present a soft system to study active oscillations in the context of soft fluidic machines. We couple an elastic membrane with a catalytic chemical reaction of liquid hydrogen peroxide that decomposes into gases. We observe that this coupling alone leads to oscillations of the reaction, and thereby of the membrane, that self‐sustain for multiple consecutive hours. By leveraging nonlinearities in the interaction between the liquid and the catalyst, the pressure oscillation does not require membranes with complex, nonlinear responses, intricate fluidic circuits, or external power sources. We show with a lumped‐parameter model that the dynamic inflation‐deflation cycle arises from an interplay between the membrane stiffness, venting resistance, and capillarity of the catalyst. Importantly, in this system, the release of energy is directly coupled to the oscillator itself. Hence, we refer to this model system as an active fluidic oscillator that embodies energy [49]. Note that we use the term embodied energy in the soft‐robotics sense, referring to energy that is physically stored within the body of the machine and directly coupled to its dynamics [49], rather than to the lifecycle‐assessment meaning of the energy required to produce a good or service.

## 2 An Active Fluidic Oscillator with Embodied Chemical Energy

To embody energy within a soft fluidic system, we consider liquid hydrogen peroxide due to its high energy density (∼2.88kJ/g), about one order of magnitude higher than common lithium‐ion batteries [30]. When the fuel (concentration of 50% hydrogen peroxide in water) comes into contact with the catalyst (99.9% pure silver wire) (Figure 1a), it rapidly decomposes the hydrogen peroxide into gas (water vapor and oxygen) and residual liquid water (Figure 1b and Movie S1), following the exothermic reaction [50]:

(1) $$2H_{2}O_{2}\left(l\right)\to 2H_{2}O\left(g,l\right)+O_{2}\left(g\right)↑,$$

where we indicate the liquid phase (l), the gas phase (g), and the released heat (↑). At atmospheric pressure, a small volume of liquid fuel decomposes into a large volume of gas, with a volume increase of ∼250 times at 50% concentration by weight in water [51]. From just 6mL of liquid fuel (Figure 1a), we can obtain ∼1.5L of gas (Figure 1b).

**FIGURE 1:**
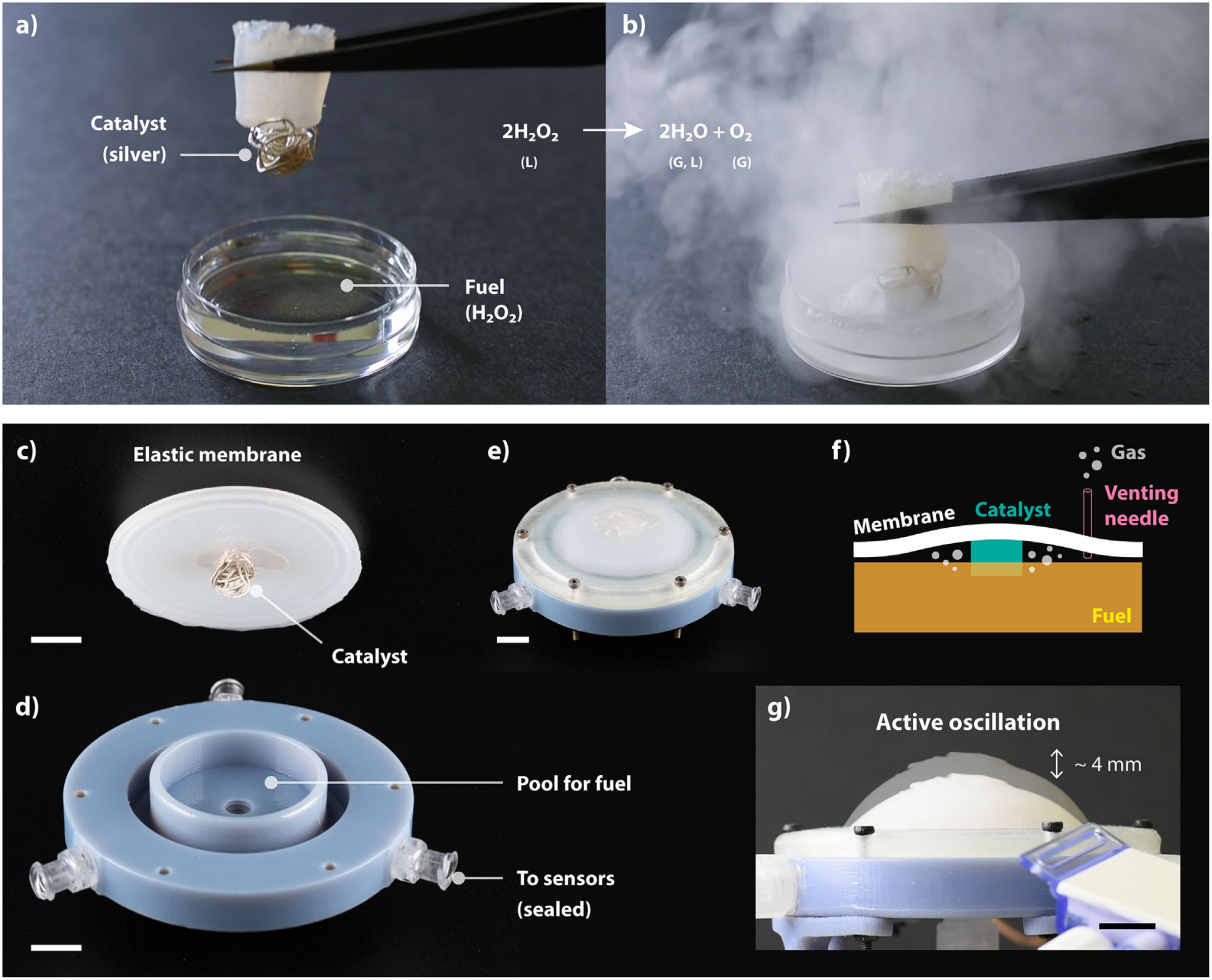
An active fluidic oscillator with embodied energy. (a) When a catalyst (50cm of silver wire with diameter 0.5mm) enters in contact with fuel (liquid hydrogen peroxide, 50% concentration in water), (b) the fuel rapidly decomposes releasing gas (water vapor and oxygen) and residual liquid water. The fuel stores energy (with energy density ∼ 1.44kJ/g, one order of magnitude higher than lithium‐ion batteries [30]) which is released in gas, and hence can be directly used to power pneumatic systems. (c) We partially embed the silver wire catalyst in an elastic membrane, leaving the majority of the surface area of the wire exposed (Supplementary Materials). (d) The fuel (6mL in volume) is contained in a 3D‐printed rigid holder, with three ports for insertion of two temperature sensors and a pressure sensor (Supplementary Materials). (e) The membrane is clamped on top of the fuel pool, with the catalyst facing the pool. (f) A needle is connected to one of the ports to vent the gases generated during the reaction. (g) The membrane exhibits active oscillations, alternatively inflating and deflating as a consequence of the reaction activating and deactivating cyclically. Scale bars in c, d, e, and g are 1cm.

With the goal of coupling the reaction directly to a soft machine, we embed the catalyst in an elastic silicone rubber membrane [Smooth‐On Dragon Skin 10 (DS10)] (Figure 1c). The catalyst is a silver wire with length 50cm and diameter 0.5mm, loosely packed in the shape of a sphere with a diameter of ∼9mm. The catalyst is partially embedded in the membrane, with the majority of the surface area of the wire left exposed (Supplementary Materials). We then 3D‐print a holder with a pool to contain 6mL of fuel, with three ports to allow for the insertion of sensors (Figure 1d). We clamp the membrane on top of this holder, with the catalyst facing the pool, obtaining a closed chamber between the membrane and the holder (Figure 1e). Lastly, we connect a needle to one of the ports and seal the others. The needle allows controlling the amount of venting of the gases generated during the reaction (Figure 1f). In Figure S4, we show a photograph of the complete experimental setup.

In an experiment where we fill the pool with fuel, we observe that this relatively simple system, composed of a flat membrane, a catalyst, fuel, and a needle (Figure 1f), spontaneously self‐sustains an active oscillation, visible from the membrane inflating and deflating cyclically (Figure 1g).

## 3 The Active Oscillations Self‐Sustain for Hours

To systematically study the active oscillation, we prepare the experiment by inflating the membrane so that the embedded catalyst is displaced away from the pool, as shown in Figure S4. This allows us to fill the pool with 6mL of fuel without triggering the reaction. After this setup procedure, we start the experiment by venting the chamber to the atmosphere through the on/off valve (Figure S4). We define the start of the experiment (t=0s) when the chamber reaches atmospheric pressure. After this initial manual interaction, we let the system evolve over time, without further external interactions.

In the first minutes of the experiment, we observe the start of an active oscillation of the membrane, which alternatively inflates and deflates between 10 and 20kPa at a frequency of ∼0.06Hz (Figure 2a and Movie S1). Interestingly, after about 2 h, the system is still oscillating, although at lower pressures (6–13kPa) and with an order‐of‐magnitude lower frequency (∼0.008Hz) (Figure 2b).

**FIGURE 2:**
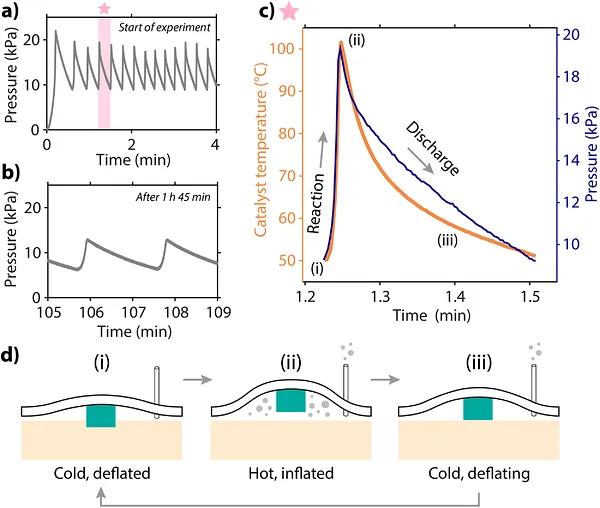
The active oscillator spontaneously inflates and deflates for hours. (a) Active oscillations occurring in the first 4 min of the experiment. (b) After 1 h and 45 min, the active oscillation is still self‐sustaining, with a lower frequency and at lower pressures. (c) The trends of the temperature at the catalyst and of the pressure during the fourth oscillation cycle (pink star in a) highlight a reaction phase followed by a discharge phase. (d Schematics of three stages of the oscillation cycle: (i) the catalyst is cold and dipped in the fuel, as the membrane is deflated, (ii) the catalyst is hot and the membrane is inflated as the reaction occurred, and (iii) the gases are venting to atmosphere, the membrane is deflating, and the catalyst is cooling down.

To better illustrate the active oscillation, we examine an individual oscillation cycle. In particular, we look at the evolution of pressure and catalyst temperature during the fourth cycle (Figure 2c). We identify two main phases within the period: a reaction phase followed by a discharge phase. At first, the catalyst temperature increases from 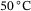 to 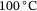, indicating the activation of the catalytic reaction (Figure 2c, orange line). This increase in temperature is accompanied by an increase in pressure to ∼20kPa (Figure 2c, blue line), as a consequence of the gases generated during the reaction. Effectively, the system is transitioning from a state with cold catalyst and deflated membrane, and the catalyst dipped in the fuel (Figure 2d‐i), to a state with hot catalyst and inflated membrane, and the catalyst displaced away from the fuel (Figure 2d‐ii). We anticipate that crucial aspects to this transition are the dipped catalyst gaining fuel at a faster rate than losing it via the reaction and the emerged catalyst holding some fuel through capillary forces even after it has lost contact with the pool. During the reaction, some of the generated gases leak into the atmosphere through the venting needle; however, the faster timescale of the reaction dominates over the slower leakage rate. We will elucidate such separations of timescales in the next sections.

When there is no more fuel held in the catalyst, the reaction ends, and pressure stops increasing (Figure 2c‐ii). This is the start of the discharge phase, in which the leakage through the venting needle takes over, causing pressure to drop (Figure 2c, blue line). Simultaneously, the absence of contact with fuel allows the catalyst to cool down (Figure 2c, orange line). As pressure decreases, the membrane deflates, causing the catalyst to displace toward the fuel (Figure 2d‐iii). Once the catalyst comes into contact with the fuel, the reaction is activated again, and the cycle repeats.

In addition to this local profile of temperature and pressure within one cycle, the active oscillations also evolve over time. In the first ∼13min (Figure 3, green star), the oscillation frequency increases from 0.06 to 0.1Hz (Figure 3a), with the maximum and minimum pressures marginally decreasing (Figure 3b). Within this period, the maximum catalyst temperature stays at around 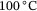, the minimum catalyst temperature rises from 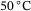 to 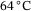 (Figure 3c), and the chamber temperature goes from 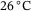 to 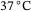 (Figure 3d).

**FIGURE 3:**
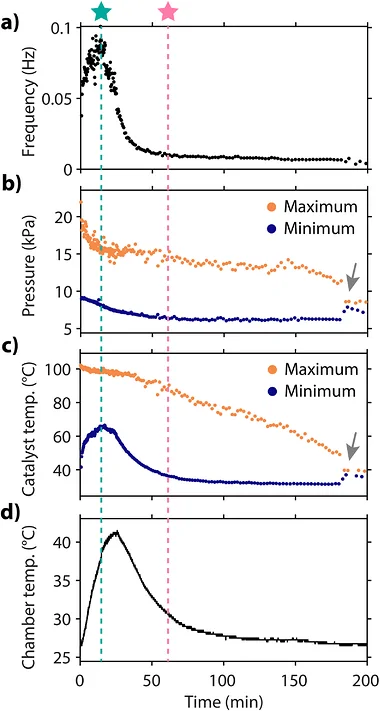
Evolution in time of the active oscillations. Evolution over more than 3 consecutive hours of (a) the oscillation frequency, (b) the maximum and minimum values of the oscillating pressure, (c) the maximum and minimum values of the oscillating catalyst temperature, and (d) the temperature inside the chamber. Grey arrows in (b) and (c) denote the transition to a small‐amplitude oscillation.

After this initial phase, the frequency starts to drop. Approximately 1h after the experiment started (Figure 3, pink star), the frequency reaches a nearly steady‐state value of ∼0.008Hz (Figure 3a). This is when the maximum and minimum values of the oscillating pressure and catalyst temperature reach a nearly steady state as well (Figure 3b,c). Excitingly, from this moment onward, the system continues to oscillate in this nearly steady‐state condition for 2 more hours. Note that we refer to it as a nearly steady‐state condition because the oscillation does not reach an actual steady state, as the fuel in the pool is being depleted in time. Eventually, at time 184min, these large‐amplitude oscillations cease to exist, and the system settles to a state with pressure ∼8kPa and catalyst temperature 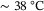 (Figure 3b,c, gray arrows).

## 4 A Lumped‐Parameter Model of the Active Oscillation

Motivated by the observation that oscillations last for hours consecutively, we further investigate the key mechanism behind the active oscillation. In particular, we know that for an active oscillation to self‐sustain, a form of positive or negative feedback [52], switching nonlinearity, or delay within the internal states of the system is required [19]. We sought to elucidate the source of the active oscillation in our specific system. We hypothesize that the active oscillation is sustained by a combination of effects. *(1)* The catalyst holds fuel via capillary forces even when it has emerged from the pool, implying that the reaction can continue even when the catalyst is not in contact with the pool surface. *(2)* The reaction occurs more slowly than the fuel wicks into the catalyst, and venting of gases to the atmosphere is slower than the rate of generation of gases, inducing an effective sequence of events with wicking first, then reaction, then venting. *(3)* The generation of reacted gases inflates the membrane, which consequently displaces the catalyst away from the fuel pool, hence acting as a negative feedback effect.

To understand the mechanism behind the active oscillation, and in particular to uncover the effects of various physical parameters on the outcome behavior, we develop a model that captures the physical contributions to the system (Figure 4 and Supplementary Materials). With the model, we aim for a qualitative, yet informative, description of the overall system. Hence, the model couples the influence of the reaction kinetics, the effects of the catalyst temperature, the capillarity of the catalyst, the mechanics of deformation of the membrane, and the leakage through the venting resistor. The model, which lumps complex physical phenomena into relatively simple relationships, qualitatively captures the experimental results and offers insights into the key phenomena that enable the active oscillation to occur. Specifically, the model teaches us that the active oscillation arises from a time offset induced by the catalyst capillarity and negative feedback resulting from the catalyst being physically attached to the inflating membrane.

**FIGURE 4:**
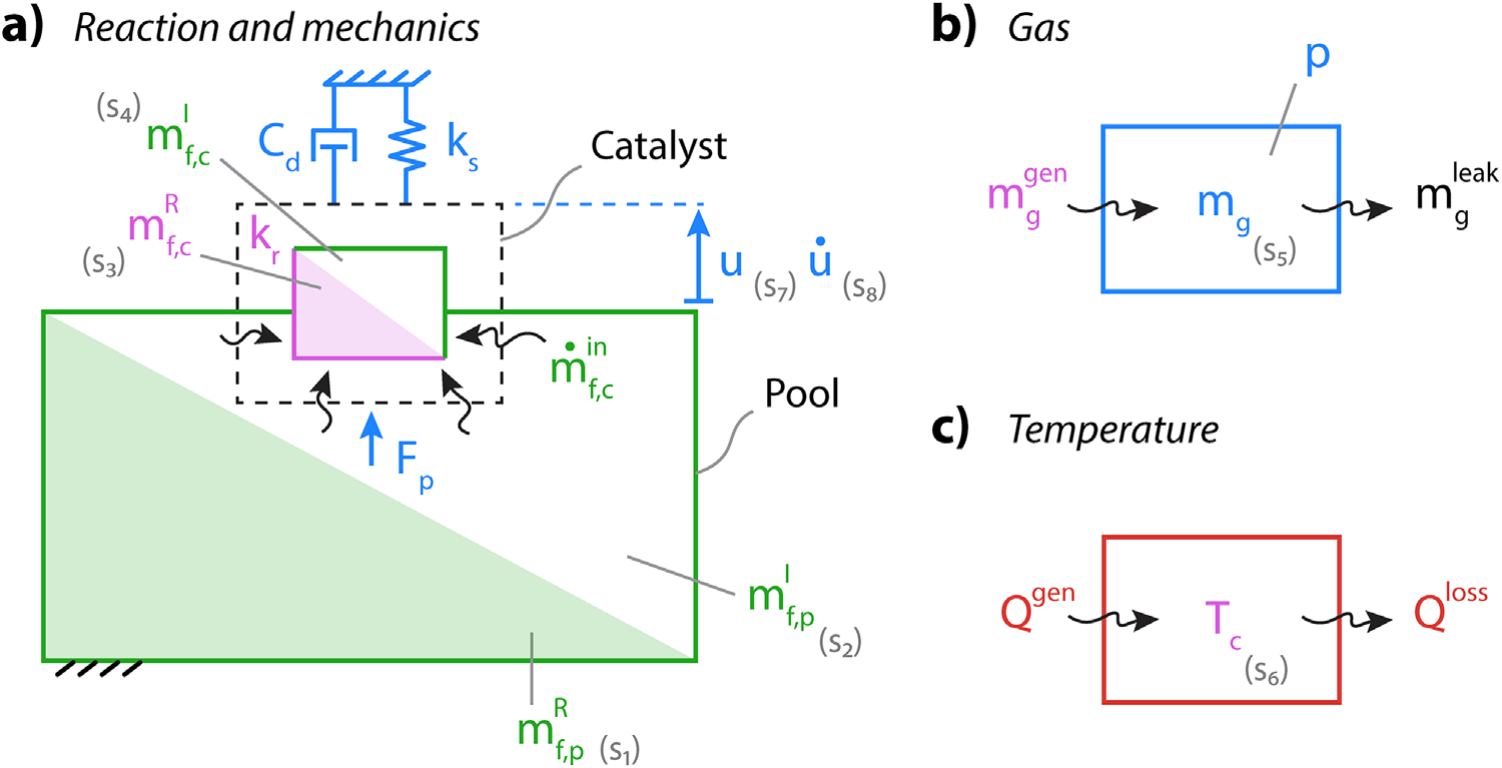
Schematic of the model of the active oscillator. (a) We model the pool as a volume of fuel with a reactable part $m_{f,p}^{R}$ (green) and an inert part $m_{f,p}^{I}$ (white). The catalyst is also modeled as a container of fuel, with a reactable part $m_{f,c}^{R}$ (purple) and an inert part $m_{f,c}^{I}$ (white). Fuel exchanges between the pool and the catalyst (black arrows), with rate ${\dot{m}}_{f,c}^{\mathrm{in}}$. Only the reactable fuel in the catalyst is assumed to decompose into gases, with a reaction rate $k_{r}$ function of the temperature of the catalyst $T_{c}$. The catalyst is attached to a linear spring with stiffness $k_{s}$ and a damper with damping coefficient $C_{d}$, and it moves upwards by a displacement u under the force $F_{p}$ generated by the pressurized gas. (b) The mass of gas generated through the reaction $m_{g}^{\text{gen}}$ increases the total mass of gas inside the chamber $m_{g}$, inducing pressurization p. The gas leaking through the venting needle $m_{g}^{\text{leak}}$ reduces the mass of gas in the chamber. (c) The temperature of the catalyst $T_{c}$ increases due to the heat $Q^{\text{gen}}$ generated by the exothermic reaction and decreases due to heat losses $Q^{\text{loss}}$. We denote the state variables as $s_{i}$, with i=1to8.

In Figure 4a, we schematically represent the subsystems of the model related to both the catalytic reaction and the mechanics of the membrane. We model the main pool as a container with fuel, of which the mass is the sum of a reactable part $m_{f,p}^{R}$ (Figure 4a, green) and an inert part $m_{f,p}^{I}$ (Figure 4a, white). This distinction comes from using a fuel made of hydrogen peroxide diluted in water: when reacting, only the hydrogen peroxide decomposes, while water does not, being inert. Note that, although Figure 4a visually represents the fuel as two distinct mass parts, the fuel is a homogeneous mixture (a solution) of hydrogen peroxide and water.

We model the catalyst as another container of fuel, with a smaller fuel capacity compared to the larger pool (Figure 4a, small rectangle). This assumption effectively implies that the catalyst can hold a certain amount of fuel through capillary forces: the catalyst acts as a small fuel container that can be replenished from the larger pool and can deplete via the reaction. Importantly, this is implicitly describing a hysteretic (hence nonlinear) interaction between fuel and membrane, for which the mass of fuel in the catalyst is not uniquely defined over the catalyst displacement. For instance, while the catalyst holds fuel when lifted and moving away from the pool, it may be empty on its way back toward the pool. The mass of fuel in the catalyst is also modeled as the sum of a reactable part $m_{f,c}^{R}$ (Figure 4a, purple) and an inert part $m_{f,c}^{I}$ (Figure 4a, white). Fuel is allowed to be exchanged between the pool and the catalyst, with an effective fuel‐uptake rate ${\dot{m}}_{f,c}^{\mathrm{in}}$ (Figure 4a, black arrows):

$${\dot{m}}_{f,c}^{\text{in}}\left(t\right)=C_{f}^{\text{eff}}\cdot A_{s}\left(t\right)\cdot \left(m_{f,c}^{\max}-m_{f,c}\left(t\right)\right).$$

with $A_{s}\left(t\right)$ the contact surface area of the catalyst with the fuel pool, $m_{f,c}\left(t\right)$ the current mass of fuel in the catalyst, $m_{f,c}^{\max}$ the maximum mass of fuel the catalyst can hold, and $C_{f}^{\mathrm{eff}}$ an effective fuel‐uptake coefficient.

Crucially, we assume that only the reactable part of fuel *in the catalyst* decomposes into gases, with a reaction rate $k_{r}$ (Figure 4a, purple). We assume that the fuel in the pool does *not* react, motivated by the experimental observation that only hydrogen peroxide in close contact with the catalyst decomposes. We assume that the mass of hydrogen peroxide attached to the silver catalyst $m_{f,c}^{R}$ decomposes into only gas mass $m_{g}^{\text{gen}}$ (water vapor and oxygen gas) without any residual liquid water. In reality, only some of the generated water evaporates due to the high temperatures around 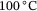 and some stays in liquid form; however, it is not obvious to quantify the amount of residual liquid water, so we neglect this contribution and we assume all water evaporates for simplicity. The hydrogen peroxide decomposition has been described as a first‐order catalytic reaction [53]:

$${\dot{m}}_{g}^{\text{gen}}\left(t\right)=k_{r}\left(T_{c}\left(t\right)\right)\cdot m_{f,c}^{R}\left(t\right).$$

This reaction is known to be temperature‐dependent, where the rate $k_{r}$ at which the reaction occurs is exponentially increasing for higher temperatures $T_{c}$ following the Arrhenius equation [54]. Since we are aiming for a qualitative (yet informative) description of the overall coupled system, for simplicity, we assume the reaction rate to be linearly increasing with the catalyst temperature $T_{c}$:

(2) $$k_{r}\left(T_{c}\left(t\right)\right)=k_{r_{0}}+C_{r}\cdot \left(T_{c}\left(t\right)-T_{0}\right),$$

with $T_{0}$ the ambient temperature, $k_{r_{0}}$ the baseline reaction rate at ambient temperature, and $C_{r}$ the reaction constant, that is the slope of the reaction rate function of catalyst temperature. In real catalytic hydrogen peroxide decomposition, the self‐accelerating Arrhenius relationship is bounded by more complex physical phenomena such as evaporative cooling [55, 56], or heat and mass transfer between the liquid and gas phases [55]. Without these additional mechanisms modeled, an Arrhenius term for reaction rate is known to risk a runaway reaction in modeling, creating a non‐physically accurate ‘explosion’ in system temperature [57]. Thus, the choice of a linear reaction rate relationship with temperature captures the necessary temperature dependence without the runaway risk, making it the more physically appropriate choice within the scope of our oscillator model.

The inflating membrane does not operate in the snap‐through [15] regime, that is, the pressure‐displacement curve is fairly linear and does not present negative stiffness regions [58, 59] in the observed deformation range. Hence, we model the membrane as a linear spring with stiffness $k_{s}$ with a damper $C_{d}$ in parallel (Figure 4a, blue). The catalyst with mass $m_{c}$ is rigidly attached to the membrane (to the bottom of the spring‐damper), and its displacement is assumed to be purely vertical. The spring compresses vertically by a displacement u (which we refer to as the catalyst displacement) under the force $F_{p}$ that originates from the pressurized gas inside the chamber. The catalyst displacement follows Newton's second law $m_{c}\cdot \ddot{u}\left(t\right)=-k_{s}\cdot u\left(t\right)-C_{d}\cdot \dot{u}\left(t\right)+F_{p}\left(t\right)$, with $F_{p}\left(t\right)=A_{m}\cdot p\left(t\right)$, where $A_{m}$ is the inner surface area of the membrane and p is the system pressure. We can neglect viscoelasticity without losing key factors underlying the active oscillation behavior, since the material's viscous dissipation plays a marginal role relative to the overall oscillation timescale (Supplementary Materials). Hence, throughout the manuscript, we assume $C_{d}=0\;Ns/m$, meaning that we approximate our membranes as ideally elastic.

The subsystem related to the internal gas is represented in Figure 4b. The reaction occurring at the catalyst generates gas products of mass $m_{g}^{\text{gen}}$, which increase the total mass of gas inside the chamber $m_{g}$, inducing pressurization p (Figure 4b, purple) under the assumption of ideal gas:

$$p\left(t\right)=\frac{m_{g}\left(t\right)}{M_{g}}\cdot \frac{R\cdot T_{0}}{V_{\text{ch}}},$$

where $M_{g}$ is the molar mass of the reacted gas, R is the gas constant, and $V_{\text{ch}}$ is the volume of the chamber. For simplicity, we assume that $V_{\mathrm{ch}}$ remains constant, though it varies in the order of magnitude of 1mL in experiments, as the membrane deforms when pressurized. The gas that leaks through the venting needle ${\dot{m}}_{g}^{\text{leak}}$ is defined as a function of pressure and a constant that represents the fluidic resistance of the venting needle $R_{l}$, as ${\dot{m}}_{g}^{\text{leak}}\left(t\right)=p\left(t\right)/R_{l}$. Intuitively, $m_{g}^{\text{leak}}$ decreases the mass of gas present in the chamber $m_{g}$, while $m_{g}^{\text{gen}}$ increases $m_{g}$ (Figure 4b, black), so ${\dot{m}}_{g}\left(t\right)={\dot{m}}_{g}^{\text{gen}}\left(t\right)-{\dot{m}}_{g}^{\text{leak}}\left(t\right)$.

The thermal subsystem of the model is shown in Figure 4c. Hydrogen peroxide decomposition is an exothermic reaction, which generates non‐negligible heat [60]. We model this phenomenon by considering that the temperature of the catalyst $T_{c}$ increases due to the heat $Q^{\text{gen}}$ generated by the exothermic reaction. The catalytic reaction generates heat as a function of the heat of decomposition of the exothermic reaction $H_{\text{dec}}$ [60] and the rate of generation of gas, following ${\dot{Q}}^{\text{gen}}\left(t\right)=H_{\text{dec}}\cdot {\dot{m}}_{g}^{\text{gen}}\left(t\right)$. In addition, $T_{c}$ decreases due to heat losses to the environment $Q^{\text{loss}}$, the rate of which is defined as ${\dot{Q}}^{\text{loss}}\left(t\right)=C_{\text{loss}}\cdot \left(T_{c}\left(t\right)-T_{0}\right)$, with a heat loss coefficient $C_{\text{loss}}$ and room temperature $T_{0}$. The temperature dynamics (Figure 4c) follow:

$${\dot{T}}_{c}\left(t\right)=\frac{{\dot{Q}}^{\text{gen}}\left(t\right)-{\dot{Q}}^{\text{loss}}\left(t\right)}{M_{C}\left(t\right)},$$

with $M_{C}\left(t\right)$ the effective heat capacity of the catalyst, which sees contributions from the silver mass $m_{c}$ and the residual mass of water present on the catalyst $m_{f,c}^{I}\left(t\right)$, following:

(3) $$M_{C}\left(t\right)=m_{c}\cdot c_{p,s}+m_{f,c}^{I}\left(t\right)\cdot c_{p,w},$$

with $c_{p,s}$ the specific heat capacity of silver and $c_{p,w}$ the specific heat capacity of water. Note that we assume that the temperature of the catalyst affects the reaction rate according to Equation (2), as the reaction is temperature‐dependent [54].

In total, our model represented in Figure 4 involves eight state variables: the mass of reactable fuel in the pool $m_{f,p}^{R}$, the mass of inert fuel in the pool $m_{f,p}^{I}$, the mass of reactable fuel in the catalyst $m_{f,c}^{R}$, the mass of inert fuel in the catalyst $m_{f,c}^{I}$, the mass of gas in the chamber $m_{g}$, the temperature of the catalyst $T_{c}$, and the displacement u and velocity $\dot{u}$ of the membrane.

In the Supplementary Materials, we report the model derivation in detail. In particular, we obtain a coupled system of eight ordinary differential equations (ODEs) in Equation S25, which we numerically integrate to simulate the active oscillation.

## 5 The Model Explains the Active Oscillation

Having developed a model that takes into account the chemical reaction, the capillarity of the catalyst, the mechanics of the membrane, and gas and temperature balances, we first aim to confirm that the model captures the active oscillation observed in experiments. We assign physically plausible values to the parameters as summarized in Table S1, where we indicate for each parameter if it is either scientifically well established, experimentally measured by us, or resulting from a qualitative fit so that the simulations qualitatively match the experiments in Figure . We integrate forward the set of ODEs in Equation S25 (initialized as in Equation S30) with the MATLAB ode23s solver, which is effective in solving stiff ODEs through a modified Rosenbrock method [61, 62]. Given the parameters in Table S1, the ODEs are stiff due to discontinuities in making or breaking contact with fuel, the varying reaction rate, and vastly differing time scales between fuel uptake, reaction rate, and gas release.

We observe that the model captures the active oscillation, with both the chamber pressure and the catalyst temperature oscillating at a frequency of ∼0.011Hz (Figure 5a), qualitatively similar to the experiments (Figure 2). The total mass of gas present in the chamber and the mass of reactable fuel in the catalyst also oscillate, with a time offset with respect to each other (Figure 5b).

**FIGURE 5:**
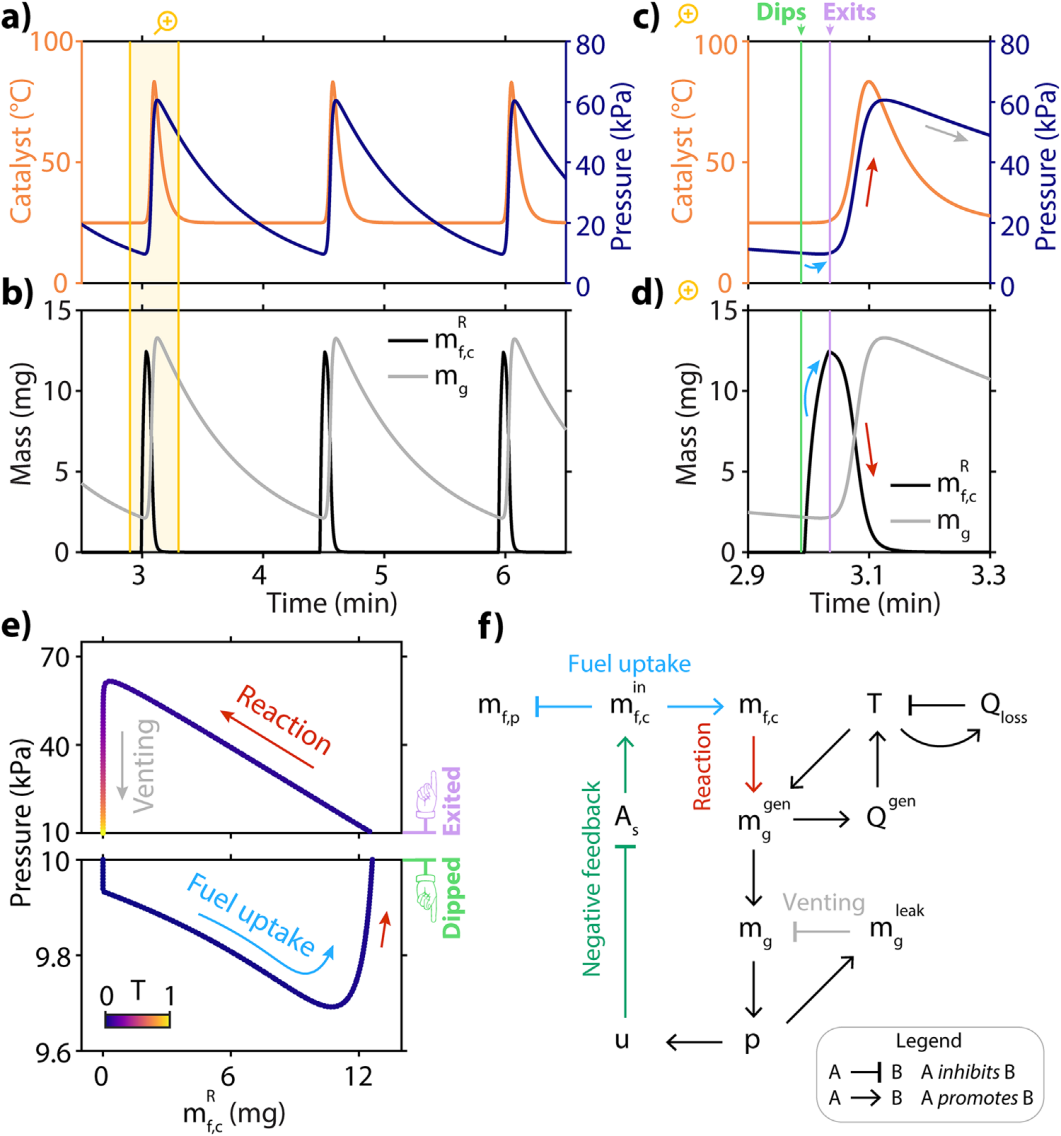
The model captures the active oscillation. (a) Evolution in time of the catalyst temperature (orange) and the chamber pressure (blue) of the modeled system (third to fifth inflation cycles). (b) Evolution in time of the mass of reactable fuel in the catalyst (black) and the mass of gas in the chamber (gray) of the modeled system (third to fifth inflation cycles). (c, d) A zoomed view of the third inflation cycle highlights when the catalyst enters the pool (green) and when the catalyst exits the pool (purple), defined as the instants when the catalyst displacement moves past the fuel pool surface (Supplementary Materials). (e) Hysteresis highlighted by a period of oscillation (T: 0 to 1) displayed in the domain of reactable fuel in the catalyst $m_{f,c}^{R}$ versus pressure. (f) We represent how the key variables in the model qualitatively affect each other. Flat arrowheads from a variable A to a variable B indicate that A inhibits B. Pointy arrowheads from variable A to variable B indicate that A promotes B. We highlight in blue the effective fuel uptake into the catalyst, causing an increase in the mass of fuel in the catalyst $m_{f,c}$. In red, we indicate the catalytic reaction that generates a mass of gas $m_{g}^{\text{gen}}$ from the mass of fuel in the catalyst $m_{f,c}$. In grey, we indicate how the gas that vents to atmosphere reduces the mass of gas in the chamber. In green, we indicate the effect of negative feedback, for which an increase in displacement u causes a decrease in the surface area of contact $A_{s}$ of the catalyst with the fuel in the pool, inhibiting fuel uptake into the catalyst. The parameters used in these simulations are listed in Table S1.

Importantly, we find that a key phenomenon enabling the active oscillation is this time offset between the catalyst gaining reactable fuel and the effective inflation of the membrane. This time offset can be better understood by looking closely at the third inflation cycle (Figure 5c,d) (avoiding the transient behavior of the first cycles). When the catalyst dips into the fuel pool (green), it gains reactable fuel via effective fuel uptake (Figure 5d, blue arrow). Pressure marginally rises because some of the gained fuel in the catalyst reacts, and importantly, it keeps rising (Figure 5c, red arrow) even after the catalyst exits the pool (purple), as a consequence of the fuel present in the catalyst reacting and turning into gases (Figure 5d, red arrow), even when the catalyst is no longer dipped in the pool.

To better understand one complete cycle, we plot pressure against reactable fuel in the catalyst $m_{f,c}^{R}$ (Figure 5e), noticing a system‐level hysteretic behavior. When dipped, $m_{f,c}^{R}$ increases while pressure drops initially (blue arrow). When the reaction picks up in the catalyst, causing pressure to increase (small red arrow), the catalyst has already gained $m_{f,c}^{R}\approx 12\;mg$. After the catalyst exits the pool, this fuel held by capillary forces continues to react, hence pressure increases while $m_{f,c}^{R}$ decreases (red arrow), until $m_{f,c}^{R}=0$. With all fuel reacted, the gas generated vents to atmosphere (grey arrow) and the catalyst cools back down (Figure 5c), bringing the system back to its initial submerged state to begin another cycle.

Hence, this model teaches us that the active oscillation arises from two sources: (1) a time offset between fuel entering the catalyst in liquid form and leaving the catalyst in the form of gases, and (2) negative feedback between pressurization and reaction. The first contribution, the time offset, is a consequence of *(1)* the catalyst being able to hold liquid on its surface through capillary forces, even when not dipped in the pool, and *(2)* the faster timescale of fuel uptake into the catalyst with respect to the timescale of the reaction, and the faster reaction than venting. Together, these effects result in a causal sequence of events, where first the fuel uptake occurs, then the reaction happens, then venting takes over. The second contribution, negative feedback, results from the catalyst being physically attached to the membrane, which displaces upward when pressurized, hence distancing the catalyst from the pool of fuel.

Notably, the slower timescale of the catalytic reaction relative to fuel delivery is also supported by our previous independent experiments with the same silver‐wire catalyst and fuel [34]. There, pressurization of a cold reactor exhibits a warm‐up delay consistent with the temperature‐dependent decomposition rate [34]. In addition, a faster fuel injection yields a less complete reaction due to reduced residence time [34]. Both these observations indicate that the fuel reaches the catalyst faster than it decomposes.

The time offset arising from the causally sequenced fuel uptake, reaction, and venting, and the negative feedback are apparent when we represent the model in a block diagram (Figure 5f) where the key variables either inhibit or promote each other, inspired by the representations often used in molecular biology [63, 64]. We notice how fuel uptake promotes the mass of fuel in the catalyst (Figure 5f, blue), which in turn promotes the generation of gases through the reaction (Figure 5f, red), which causes pressurization and promotes the catalyst displacement u to increase. In turn, the catalyst displacing upwards causes a decrease in the surface area of contact $A_{s}$ of the catalyst with the fuel in the pool, inhibiting fuel uptake to the catalyst, thereby acting as a negative feedback mechanism (Figure 5f, green). Looking at the phase diagram in Figure S7, where we analyze the system's response to positive and negative feedback strengths, it is evident that the active oscillation is sustained when the negative feedback effects balance the positive feedback effects.

In addition to explaining the active oscillation behavior, the model also captures the long‐term evolution of the oscillation in Figure 3. In particular, the model captures the decrease of the maximum and minimum oscillation pressures in time (Figure S7a, blue). This is a consequence of the available reactable fuel in the pool being depleted, as indicated by the concentration of fuel in the pool dropping from $\alpha_{p}=0.42$ to $\alpha_{p}=0.07$ over the course of 8 simulated hours (Figure S8a7a, dark red). The reduced amount of available reactable fuel effectively results in the system reacting less and less, as indicated by the maximum catalyst temperature dropping to ambient temperature (Figure S8b). When this condition is reached after approximately 8 hours, the time scale of gas generation is no longer shorter than that of gas venting, and the system stops oscillating, as also observed in experiments (Figure 3).

## 6 Influence of Parameters on the Active Oscillation

Having learned that the separation of timescales and the negative feedback intrinsic to our system are the sources of the active oscillation, we wonder how the system responds to variations in key physical parameters. In particular, we vary parameters that can be tuned by design, such as membrane stiffness and venting resistance, to confirm that the model captures a change of behavior that can be observed experimentally. Then, we investigate how the behavior of the modeled system changes when we vary the parameters intrinsic to the reaction, such as catalyst capillary capacity and reaction constant. These two studies inform us whether there are other regimes that the system can be placed in (in addition to the already observed oscillation) and whether we can tune specific parameters to obtain desired output behaviors, such as oscillation frequency and amplitude.

### 6.1 Influence of Design Parameters

Among the various parameters in Table S1, we focus on the membrane stiffness $k_{s}$ and the venting resistance $R_{l}$ as key design parameters, because they are relatively easy to tune by a designer. The former can be increased by casting the membrane with a stiffer silicone, while the latter can be tuned by swapping the venting needle with one with a different Gauge value, that is, with a different inner diameter.

When varying the membrane stiffness $k_{s}$ by four orders of magnitude, we visually identify three distinct regimes (Figure 6a). The first regime shows that a soft membrane suppresses large oscillations after an initial inflation (Figure 6b‐i). In this regime, the membrane stiffness is so low that even small pressure changes cause large displacements. Hence, as soon as the reaction generates even a small amount of gas, the membrane rapidly lifts the catalyst past the pool surface. This effect induces small oscillations near the zero‐pressure condition, with the catalyst remaining close to the pool surface. In the second regime, the frequency of oscillation increases when the membrane is stiffer, while at the same time, the maximum reached pressure increases as well (Figure 6b‐ii, iii). The increase in frequency is a consequence of the discharge phase occurring within a short time period, as the stiffer membrane leads to a smaller upward displacement of the catalyst from the fuel pool. The higher pressure is a direct result of the increased stiffness, as the displacement for which the catalyst lifts past the pool occurs at a higher pressure. For even stiffer membranes, the system operates in the third regime, where the catalyst is dipped in the fuel and the reaction keeps occurring without oscillations, until the fuel in the pool completely depletes (Figure 6b‐iv). The stiff‐membrane regime in Figure 6b‐iv differs from the soft‐membrane regime in Figure 6b‐i, in that a stiffer membrane forces the catalyst to fully dip in the pool and induce a high‐temperature, high‐pressure reaction, while a softer membrane deforms considerably even at low pressure (at slow reaction rates), leading to a low‐temperature, low‐pressure reaction where the catalyst stays on the surface of the pool.

**FIGURE 6:**
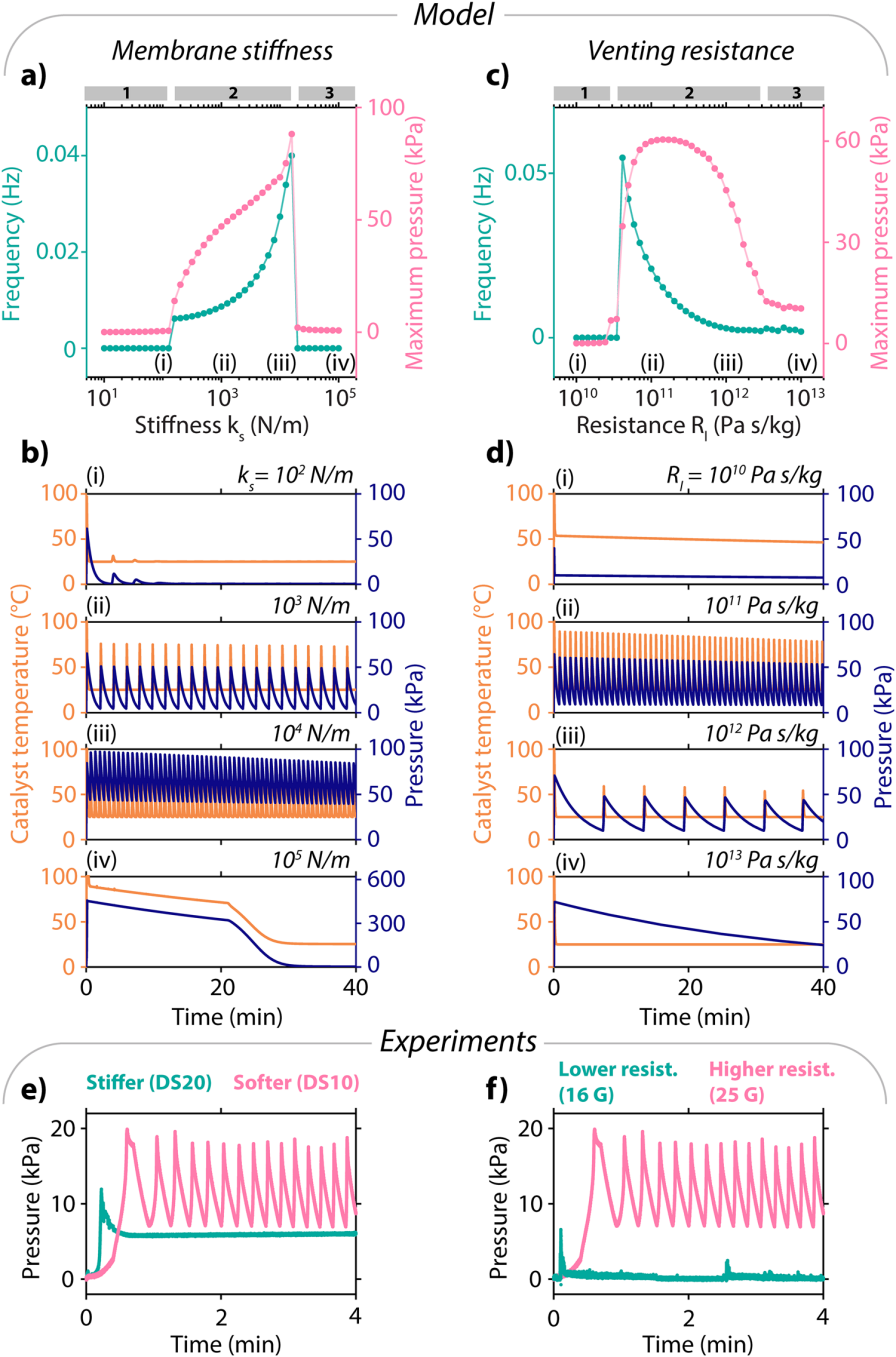
Influence of membrane stiffness and venting resistance on the active oscillation. (a) Frequency (green) and maximum pressure (pink) of the active oscillation when varying the membrane stiffness $k_{s}$ from $1\times 10^{1}\;N/m$ to $1\times 10^{5}\;N/m$. The three regimes are highlighted with grey bars, with numbers 1, 2, and 3. (b) Catalyst temperature (orange) and pressure (blue) for the four key cases (i–iv) in (a). (c) Frequency (green) and maximum pressure (pink) of the active oscillation when varying the venting resistance $R_{l}$ from $1\times 10^{10}\;\mathrm{Pa}s/\mathrm{kg}$ to $1\times 10^{13}\;\mathrm{Pa}s/\mathrm{kg}$. The three regimes are highlighted with grey bars, with numbers 1, 2, and 3. (d) Catalyst temperature (orange) and pressure (blue) for the four key cases (i–iv) in c. All simulation parameters (apart from the varied ones) are listed in Table S1. When frequency is zero, we define the maximum pressure as the pressure value at the end of the test, at time 40min. (e) Experimental evolution in time of the pressure in the active oscillator with a stiffer membrane with Young's modulus ∼0.621MPa (Smooth‐On DS20) (green) and with a softer membrane with Young's modulus ∼0.237MPa (Smooth‐On DS10) (pink), given the same venting resistance (25 G needle). f Experimental evolution in time of the pressure in the active oscillator with a lower venting resistance (16 G needle) (green) and with a higher venting resistance (25 G needle) (pink), given the same membrane (Smooth‐On DS10). Pink datapoints in e and f are the same experiment (DS10 membrane and 25 G needle).

When varying the venting resistance $R_{l}$, too, we observe three operational regimes (Figure 6c). In the first regime, the resistance is so low that the reacted gases vent to atmosphere without inducing pressurization in the chamber (Figure 6d‐i), as the venting timescale is in the same order of the reaction timescale. In the second regime, for low venting resistance, we observe relatively high oscillation frequencies, as the discharge phase occurs relatively rapidly (Figure 6d‐ii). For higher venting resistance, the maximum reached pressure decreases (Figure 6c, pink). This reduction is a result of the diminished losses of the reacted gases through venting: for each oscillation cycle, the reacted gases more effectively displace the membrane upward, stopping the reaction earlier. Simultaneously, for higher venting resistance, the frequency drops (Figure 6c, green) because the discharge timescale lengthens (Figure 6d‐ii, ‐iii). Lastly, in the third regime, too high a venting resistance effectively suppresses subsequent oscillations after a first one, as after 70 min the membrane settles in a position where the catalyst is in contact with the pool surface (∼10kPa) (Figure 6d‐iv). This settling at the surface is a consequence of the rapid lifting of the membrane by small amounts of generated gas, which shortens the dipping time below the fuel uptake timescale, hence inducing a small amount of fuel to be captured by the catalyst during the dipping time. From our numerical simulations, we extract the captured reactable mass of fuel in the catalyst in this regime, obtaining $m_{f,c}^{R}\approx 1\;mg$: an order of magnitude lower compared to the >10mg of the typical large oscillations as in Figure 5. Looking at the schematic in Figure 5f, we understand how such changes in venting resistance affect the behavior of the system. Changing $R_{l}$ affects the lower loop in the schematic (Figure 5f, grey), inducing too low or too high pressure p, which consequently reflects in the amount of negative feedback that displacement u will induce, eventually inhibiting oscillations.

We check that such dependencies on parameters predicted by the model are aligned with experimental results. First, we compare membranes made with distinct materials: a stiffer one made of DS20 with Young's modulus ∼0.621MPa, and the softer one that we used so far made of DS10 with Young's modulus ∼0.237MPa. We derived these values of Young's moduli as E=3μ, valid given the incompressibility of these elastomers, with μ the Neo‐Hookean shear moduli reported in [65]. When we equip the active oscillator with the stiffer DS20 membrane instead of the previously used DS10, we do not observe an oscillation anymore. Instead, we observe an initial increase in pressure (Figure 6e, green line), symptomatic of the reaction occurring. We then observe a gradual decrease in pressure, which converges within 15min to a static condition with ∼7.5kPa. This non‐oscillatory condition is qualitatively similar to the predicted case with high spring stiffness in Figure 6b‐iv. In contrast, the system with a softer membrane displays oscillations (Figure 6e, pink line). Similarly to varying the membrane stiffness, we check how the system responds to variations in the venting resistance. We swap the 25 G venting needle with a needle with a larger diameter (16 G), which offers lower resistance to airflow (Figure 6f). We observe the membrane initially inflating to ∼5kPa, and then rapidly going back to atmospheric pressure (0kPa). Although the reaction is occurring, the low venting resistance results in the generated gases venting to atmosphere without pressurizing the chamber. Since the membrane does not inflate, the catalyst stays dipped in the fuel, constantly inducing the reaction. Hence, the system stays at a state with zero pressure (Figure 6f, green line) and high catalyst temperature around 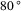. A lower venting resistance suppressing oscillations is in line with the prediction of the model (Figure 6d‐i).

These results indicate that the membrane stiffness and the venting resistance, contextualized in the coupled system with intrinsic negative feedback and internal time offset, both enable the active oscillation to arise, and can be leveraged as design tools to tune the oscillation performance. Importantly, the results from the simulations qualitatively match the experiments, where we observed that both a stiffer membrane and a lower resistance suppress oscillations.

### 6.2 Influence of Capillarity and Reaction Parameters

We now explore how the system behaves when the capillarity of the catalyst has a weaker or stronger effect, that is, when the catalyst can hold more or less volume of fuel on its surface through capillary forces. We observe three main regimes when we vary the maximum volume of fuel that the catalyst can hold ($V_{f,c}^{\max}$) by 3 orders of magnitude, between 3μL and 3mL (Figure 7a). For a very low volume of 3μL, there is no oscillation (Figure 7a, grey bar ‘1'). The second regime appears between 3μL and 0.3mL (Figure 7a, grey bar ‘2'). In this regime, the maximum pressure increases (Figure 7a, pink) and the frequency decreases (Figure 7a, green) for larger capillary volumes. This trend is expected from the assumption that all the reactable fuel present on the catalyst must react. Hence, larger volumes of fuel on the catalyst will result in larger inflation, increasing the amplitude and lengthening the oscillation period (Figure 7b(ii, iii)). The third regime between 0.3mL and 3mL shows the maximum pressure dropping for increasing capillary volume, and the frequency even reaching zero for high enough volume values around 3mL (Figure 7a, grey bar ‘3'). In this regime, the catalyst can hold so much fuel that the first oscillation cycle substantially depletes the pool of fuel (which has an initial volume of 6mL). This depletion in the first cycle results in the subsequent cycles reaching lower maximum pressures. If the fuel volume in the catalyst is large enough, the first cycle depletes the pool to a point that subsequent oscillations do not occur within the tested time frame (Figure 7b‐iv).

**FIGURE 7:**
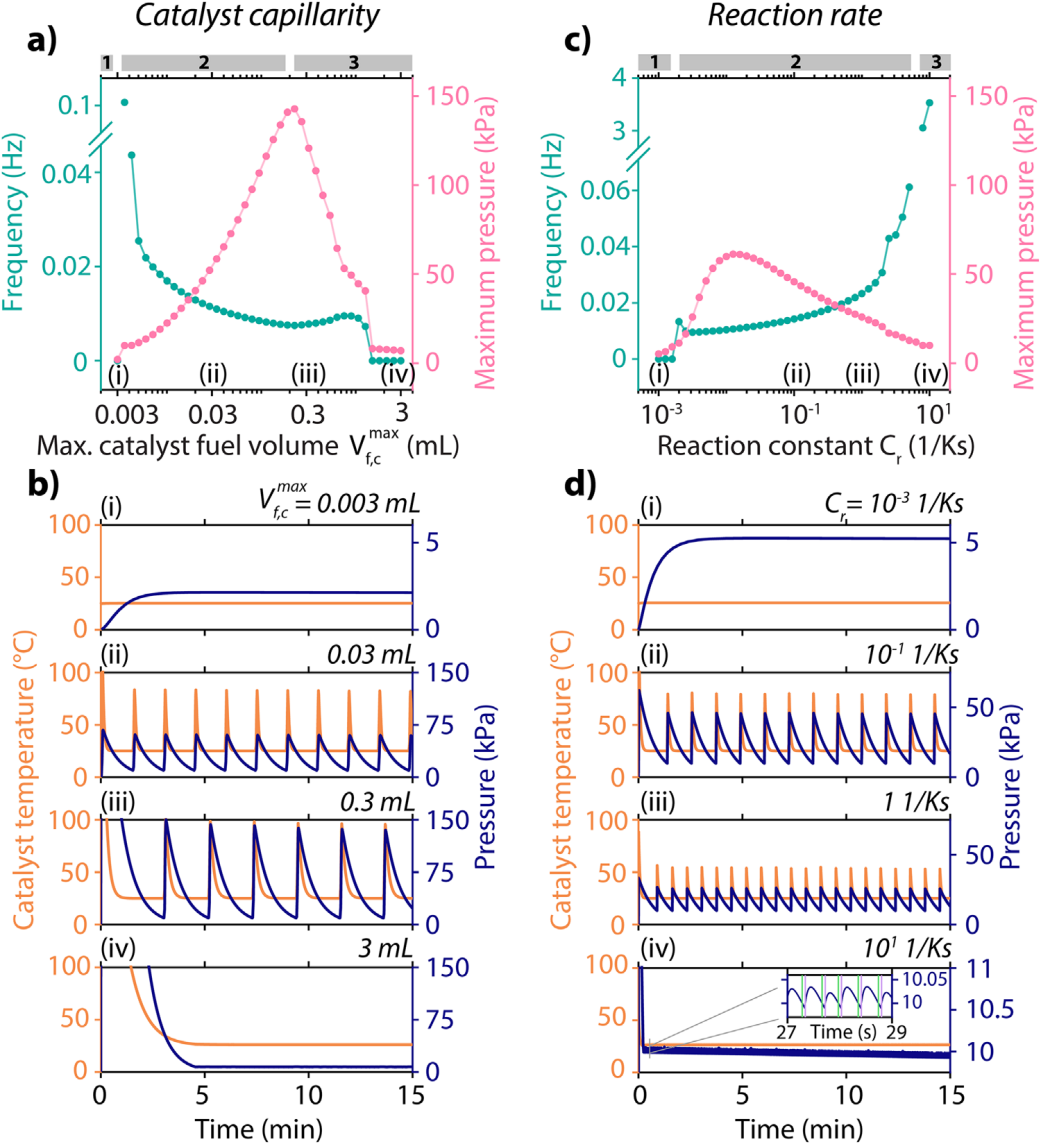
Influence of catalyst capillarity and reaction constant on the active oscillation. (a) Frequency (green) and maximum pressure (pink) of the active oscillation when varying the maximum volume of fuel in the catalyst $V_{f,c\max}$ from 3μL to 3mL. The three regimes are highlighted with grey bars, with numbers 1, 2, and 3. (b) Catalyst temperature (orange) and pressure (blue) for the four key cases (i–iv) in (a). (c) Frequency (green) and maximum pressure (pink) of the active oscillation when varying the reaction constant $C_{r}$ from $1\times 10^{-3}\;1/Ks$ to $1\times 10^{1}\;1/Ks$. The three regimes are highlighted with grey bars, with numbers 1, 2, and 3. (d) Catalyst temperature (orange) and pressure (blue) for the four key cases (i–iv) in (c). The inset in (iv) highlights the high‐frequency oscillation occurring over 2s, with the catalyst oscillating at low amplitude around the pool level, rapidly entering (green) and exiting (purple) the pool. All simulation parameters (apart from the varied ones) are listed in Table S1.

Note that the regimes ‘1’ and ‘3’ are not physically relevant to our specific experiment, where the actual catalyst can hold around 0.05mL of liquid through capillary forces (Supplementary Materials). However, looking at such extremes teaches us that *(1)* sufficient capillary volume is required for a limit cycle to appear, otherwise the small amount of fuel held by the catalyst would react before displacing past the pool surface, and *(2)* too large a capillary volume would inhibit the oscillation after one initial inflation.

We then investigate the influence of the rate of the reaction on the active oscillation. When varying the reaction constant $C_{r}$ (that is, the slope of the reaction rate function of temperature in Equation (2)) by four orders of magnitude, we visually identify three main regimes as well (Figure 7c).

The first regime appears for very low values of the reaction constant around $C_{r}=1\times 10^{-3}\;1/Ks$ (Figure 7c, grey bar ‘1'). This regime corresponds to a reaction rate so low that the reacted fuel is replenished through fuel uptake before the catalyst can lift past the pool surface, while the generated gases directly leak through the venting needle: the system stabilizes around 5kPa of pressure and with the catalyst at ambient temperature (Figure 7d‐i).

The second regime (Figure 7c, grey bar ‘2') shows an increase in oscillation frequency (green) for higher values of the reaction constant. The maximum pressure (pink) first increases for reaction constant values up to $1\times 10^{-2}\;1/Ks$. This is a consequence of a higher reaction rate, which in turn causes an increase in the catalyst temperature, which in turn affects the reaction rate itself. Overall, the fuel reacts more rapidly, thereby gas generation dominates over the gas losses through the venting needle. Beyond this reaction constant value around $1\times 10^{-2}\;1/Ks$, we observe a decrease in the maximum pressure. This reduced maximum pressure for higher $C_{r}$ is a consequence of the higher timescale of the reaction with respect to the fuel uptake, which implies that the catalyst does not have enough time to fully replenish its local fuel reservoir with reactable fuel. For instance, by increasing $C_{r}$ from $1\times 10^{-1}\;1/Ks$ to 11/Ks (cases (ii) and (iii) in Figure 7d), the results of the simulations for the maximum reactable fuel mass in the catalyst show that it lowers from ∼8mg to ∼3.5mg. As a consequence, the catalyst has a lower concentration of fuel, and hence the reaction will generate fewer gases, inducing less pressure inside the chamber.

The third regime (Figure 7c, grey bar ‘3') occurs at relatively high values of reaction constant around 101/Ks. In these conditions, the reaction timescale is much faster than the fuel‐uptake timescale, and the system oscillates at higher frequencies above 3Hz, at a pressure that stays around 10kPa. Because the reaction timescale is faster than the fuel‐uptake timescale, even relatively small amounts of liquid fuel captured by the catalyst get rapidly converted to gases, generating a relatively small amount of gas which minimally inflates the membrane. Hence, the system is not undertaking the large‐amplitude oscillations observed in the other regimes, and it rapidly exits and re‐enters the pool of fuel (Figure 7d‐iv, inset), effectively settling to this partially inflated condition with the catalyst displacing around the contact point with the pool surface.

Hence, we learned that if the reaction is not fast enough, oscillations do not occur, and the catalyst stays fully dipped in the pool (Figure 7d‐i). On the other hand, too fast a reaction results in the system oscillating with relatively small amplitude and high frequency around the surface of the pool (Figure 7d‐iv), without large‐amplitude oscillations that could be meaningfully harnessed for soft robotics applications.

## 7 Discussion

We introduced a system that undergoes active oscillations by harnessing the coupling between the deformation of an elastic membrane and a catalytic reaction. We observe oscillations in which the access to and release of energy are directly coupled to internal states of the system, effectively embodying the energy source within the oscillator itself. Through a lumped‐parameter model, we learned that the key phenomena underlying the oscillation are the negative feedback resulting from the catalyst being attached to the deforming membrane and the internal time offsets between fuel uptake, reaction, and venting, which arise from the three different timescales.

The model qualitatively captures the oscillation dynamics, and it reproduces the experimental results when varying parameters. Hence, the model both enables the understanding of the phenomena at play and can prove useful to provide design guidelines for oscillating soft robots. However, the current model follows a number of simplifications, which in future work can be extended to capture more details of the experiments. For instance, we model the reaction rate as linearly dependent on the catalyst temperature, while in reality, an exponential trend is expected [54]. Moreover, we assume that the chamber stays at ambient temperature, while experiments show that it can rise (Figure 3d). This assumption is the likely cause of the increase in frequency at the beginning of the experiment (Figure 3a), which our model does not capture: the model only describes the quasi‐steady‐state of the system, failing at capturing the initial transient. Our simplifications also account for the main quantitative discrepancies between model and experiment. The model overestimates pressure (Figure S9) mainly because we assume that all reaction water evaporates, so the generated gas comprises both oxygen and water vapor (up to three moles of gas per two moles of hydrogen peroxide); experimentally, a fraction of the water stays liquid, lowering the gas mass and the pressure. The constant‐chamber‐volume assumption, which neglects the pressure relief from membrane inflation, also contributes to overestimating pressure. Conversely, the model underestimates the minimum catalyst temperature (Figure S8b), because we fix the heat sink at ambient temperature in the heat‐loss term (Equation S18), whereas in reality the chamber heats up (Figure 3), thereby flooring the catalyst at a higher minimum. Because the model parameters were fitted to qualitatively reproduce the early‐time oscillation of Figure 2 rather than the magnitudes of Figure 3 (Table S1), it is best regarded as a qualitative tool to reveal underlying mechanisms. For quantitatively accurate, application‐specific design, the lumped description could be complemented by higher‐fidelity coupled thermo‐chemo‐mechanical numerical simulations that resolve membrane mechanics, Arrhenius kinetics, wicking, water phase change, and the spatial temperature field.

Our model allowed for a prediction of the behavior of the oscillator when the catalyst capillarity and the reaction rate are varied (Figure 7). Although outside the scope of this work, future research could confirm these trends experimentally. In fact, the reaction constant could be varied experimentally by using fuel with a lower or higher concentration of hydrogen peroxide, or by adding sodium hydroxide to the 50% concentrated fuel, to change the pH and obtain a more reactive fuel. Moreover, custom manufacturing of the silver catalyst would allow fine‐tuning the amount of fuel that can be held through capillarity.

The current design of the active oscillator serves the purpose of an experimental testbed that operates under controlled conditions, so that we can effectively isolate the various contributions and understand the phenomena at play. For functional operation in practical real‐world applications, we expect that the current prototype will require several changes. The device now relies on gravity to contain the fuel in the pool; other fuel storage solutions, such as soft porous materials like sponges [66, 67], would allow orientation‐independent operation. Moreover, the fabrication of the catalyst now follows a largely manual procedure (Supplementary Materials), hindering scalability and repeatability. Other manufacturing methods should be considered, such as casting or machining silver. By finely controlling the catalyst porosity at the fabrication stage, one could be able to derive a quantitative prediction of the detailed wicking dynamics [68] of the fuel within the porous catalyst, rather than resorting to an effective fuel‐uptake term as we did here. In addition, we observed relatively limited membrane displacement of ∼4mm upon oscillation (Figure 1g). Tuning design parameters (such as the membrane design) to obtain higher pressures (Figure 6) and, hence, higher displacements, will be relevant in future works aimed at soft robotic applications.

For practical implementations, we note that the initial one‐hour transient (Figure 3) does not correspond to a non‐operational start‐up time, since the system is already actively oscillating during this period. Rather, the transient reflects the slow evolution of internal states such as chamber temperature. For applications requiring a prescribed frequency or amplitude from the start, future work could introduce additional physical feedback mechanisms to stabilize operation around a desired limit cycle.

The relatively‐low‐frequency regime of our oscillator (0.008Hzto0.1Hz) suggests two routes toward soft robotic applications. Directly, it is suited to tasks that call for slow, sustained cyclic actuation without electronics, such as peristaltic and crawling locomotion, for which comparable electronics‐free systems operate at similar rates of a fraction of a hertz [29, 69]. Where faster actuation is instead required, the oscillator could act as a high‐level oscillatory power supply within a hierarchical architecture, in which the slow active oscillation feeds faster passive fluidic oscillators downstream, such as relaxation [23, 26], ring [28], or kinking [20] oscillators, which generate high‐frequency signals.

Despite embodying energy, the proposed approach to achieve active oscillations in soft robotic systems consumes fuel in the process. Although we showed that 6mL of fuel is enough for the oscillations to sustain for more than 3 h consecutively, considerably longer than the minutes typical of pressurized canisters [29], our approach opens the question of refueling. Refueling would involve transportation of hydrogen peroxide in bulk from the manufacturing plant, where it is currently manufactured with energy‐intensive and centralized processes [70], to the site of operation of the robot. Besides being an impractical solution to the goal of energy‐autonomous robots, transportation, on‐site storage, and handling of hydrogen peroxide are hazardous, requiring the implementation of specialized storage and handling infrastructure in any facility wishing to use this technology [71, 72]. This would be a practical challenge to adoption, which may be softened through the future development of robotics‐friendly hydrogen peroxide devices and infrastructure, similar to the existing technology developed around other hazardous energy sources, such as lithium‐ion batteries or gasoline. In a real‐world scenario, where an untethered soft robot operates autonomously in the field, we envision a local approach to the synthesis of hydrogen peroxide directly from the water and air that are available in the surrounding environment. This approach has proven to be within reach, as researchers obtained concentrations of hydrogen peroxide up to 20% from water and oxygen through direct electrosynthesis [73]. However, considerable research effort is still required to obtain increased concentrations and to devise synthesis mechanisms that are embodied in the machine itself.

With this preliminary and exploratory work, we show that relatively simple mechanical structures, such as flat elastic membranes, can undergo active oscillations when carefully coupled to other phenomena, such as capillary action and catalytic reactions. As such, we expand the library of phenomena that can be harnessed to achieve self‐oscillations in soft fluidic machines, providing additional routes to physically control soft robots while embodying energy.

## Funding

J.T.B.O. acknowledges the European Union's 2020 ERC‐STG under grant agreement No. 948132. S.D.G. acknowledges the MathWorks Fellowship. E.T.R. acknowledges the MIT MechE Department and IMES. This work is part of the Dutch Research Council (NWO) and was performed at Massachusetts Institute of Technology and at AMOLF.

## Conflicts of Interest

The authors declares no conflicts of interest.

## Supporting information


**Supporting File 1**: advs77877‐sup‐0001‐SuppMat.pdf.

**Supporting File 2**: advs77877‐sup‐0002‐VideoS1.mp4.

## Data Availability

The data that support the findings of this study are available from the corresponding author upon reasonable request.
