# Supplementary material for: Active Oscillations of Elastic Membranes Arising from the Coupling with a Catalytic Reaction
Source: Adv Sci (Weinh). 2026 Oct 8:e77877. Online ahead of print. doi: 10.1002/advs.77877 (PMC13650050; doi:10.1002/advs.77877)
Supplement: Supplementary file 1 [file ADVS-9999-e77877-s001.pdf]

# Supplementary Materials

## Active Oscillations of Elastic Membranes Arising from the Coupling with a Catalytic Reaction

*Alberto Comoretto Samuel D. Gollob Erik Steur Kaitlyn P. Becker Ellen T. Roche* Johannes T.B. Overvelde**

## 1 Manufacturing the active oscillator

To build the catalyst, we take a length of 50 cm of 99.9 % pure silver wire with diameter 0.5 mm (Sigma-Aldrich®, 327026). We manually deform the wire and coil it on itself, until it is loosely packed in the shape of a sphere with a diameter of $\sim 9$ mm. To avoid contamination of the catalyst, we manipulate the wire with clean tweezers. While coiling, we make sure not to tighten the wire, so that most of the wire surface area is exposed. During the coiling process, we create two loops external to the sphere (Fig. S1a), to ease embedding in the silicone membrane in the next step.
We attach the catalyst to a 3D-printed mold. The mold has the negative shape of the membrane, with a suspension block at the center. The catalyst is placed over the suspension block so that the two loops of the catalyst will be embedded in the poured silicone (Fig. S1b). A thin polypropylene thread (diameter $\sim 0.1$ mm) is looped around the catalyst and through two holes in the mold to keep the catalyst in place while pouring the silicone (Fig. S1b,C). We then pour silicone (pre-mixed part A and part B) into the open mold, and we let it cure (Fig. S1d). After curing, we cut the polypropylene thread and remove it. The result is a silicone membrane with the catalyst partially embedded. The two external loops of the catalyst are fully embedded for a secure attachment to the membrane (Fig. S2a). Due to the presence of the suspension block in the mold, the membrane is not airtight on the bottom side (Fig. S2b). To seal the membrane, we deposit silicone adhesive (Sil-Poxy™, Smooth-On) over the hole left by the suspension block (Fig. S2c).
In Figure S3, we show the cross-section of the complete active oscillator introduced in Figure 1, with the membrane suspended over the 3D-printed pool that contains the fuel. The fuel is an aqueous solution of 50% hydrogen peroxide (Evonik Inc. PROPULSE HTP, 50% by weight). The pool (Fig. S3, bottom) is 3D-printed with a Stratasys Objet30 Prime, in the material VeroBlue (RGD840). The holder clamp (Fig. S3, top) is 3D-printed using a Formlabs Form 3B, in the material Clear Resin V4.

## 2 Experimental setup

To acquire pressure, we use a Pendotech Luer single-use pressure sensor (PREPS-N-000), with the acquisition board PowerLab 16/35 (ADInstruments). To acquire temperature, we use two bare-wire bead-probe thermocouples that can measure air or surface temperatures (Type-K Glass Braid Insulated, Adafruit), each with an amplifier board (MAX31855 breakout board, Adafruit), connected to a microcontroller (METRO 328, Adafruit). We directly attach one thermocouple to the catalyst, and we suspend the other thermocouple mid-air inside the chamber. In Figures S4 and S5, we report photographs of the complete experimental setup, showing the connections with the pressure sensor and the two thermocouples.
Each experiment involves data acquisition of pressure inside the chamber and of the two temperatures at the catalyst and inside the chamber, while the device oscillates. Each experiment is preceded by the following steps.

1. We place the on/off valve (Fig. S4) so that the chamber is not fluidically connected to the pressure sensor and the venting needle.
2. By using a syringe, we inflate the membrane via the one-way valve at the bottom of the holder, until the catalyst is above the surface of fuel in the pool.

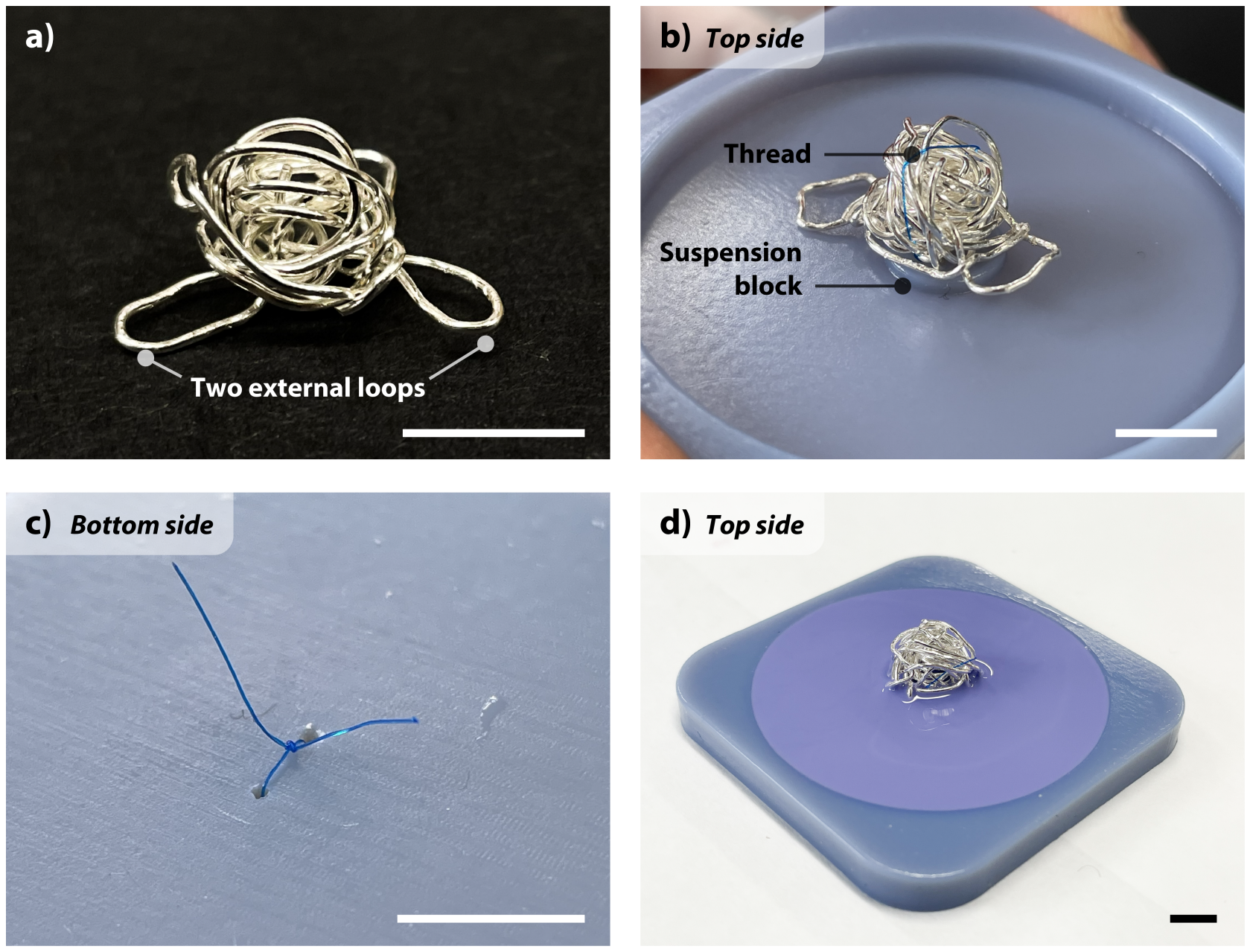


Figure S1: **Manufacturing of the membrane with the embedded catalyst.** (**a**) We build the catalyst by manipulating silver wire (0.5 mm diameter) in the shape of a sphere, with two external loops. (**b**) We connect the catalyst to the mold, over a suspension block, using a thin polypropylene thread. (**c**) The polypropylene thread is tied on the bottom side of the mold. (**d**) We pour silicone into the open mold. All scale bars are 1 cm.

3. We inject 6 mL of fuel through the same one-way valve.
4. We start acquiring temperature and pressure data.
5. We rotate the valve counterclockwise (in relation to Figure S4) to connect the chamber to both the pressure sensor and the venting needle. The membrane will deflate until the catalyst touches the fuel, and the experiment starts.

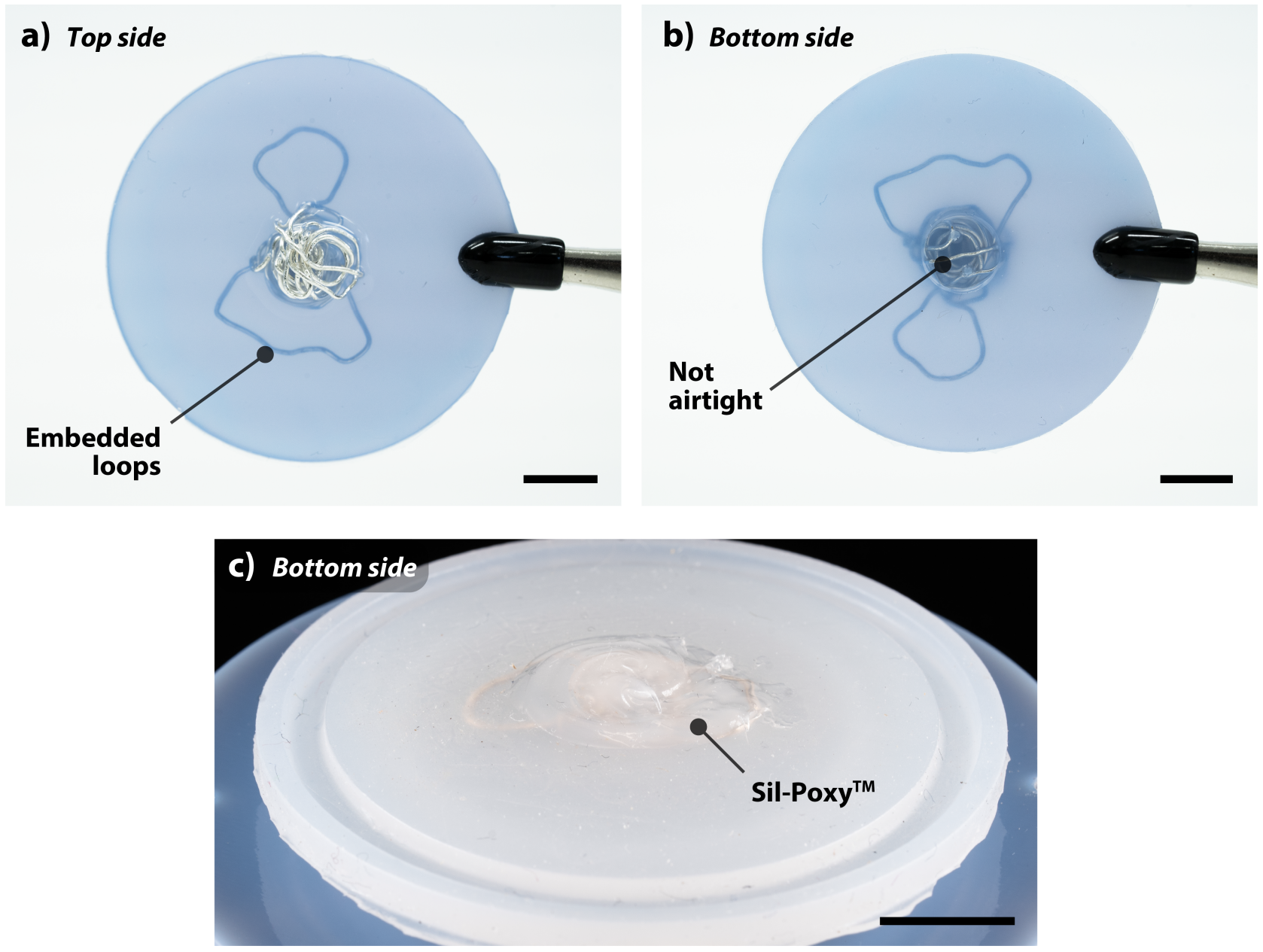


Figure S2: **Sealing the membrane.** (**a**) Top view of the membrane with the catalyst. The embedded loops are visible inside the semi-transparent silicone. (**b**) The bottom view of the membrane after demolding highlights the area around the suspension block not being airtight. (**c**) We deposit Sil-Poxy$^{TM}$ over the hole left by the suspension block. Note that the bottom side of the membrane, as indicated here, will be the top side of the membrane when clamped in the final device. All scale bars are 1 cm.

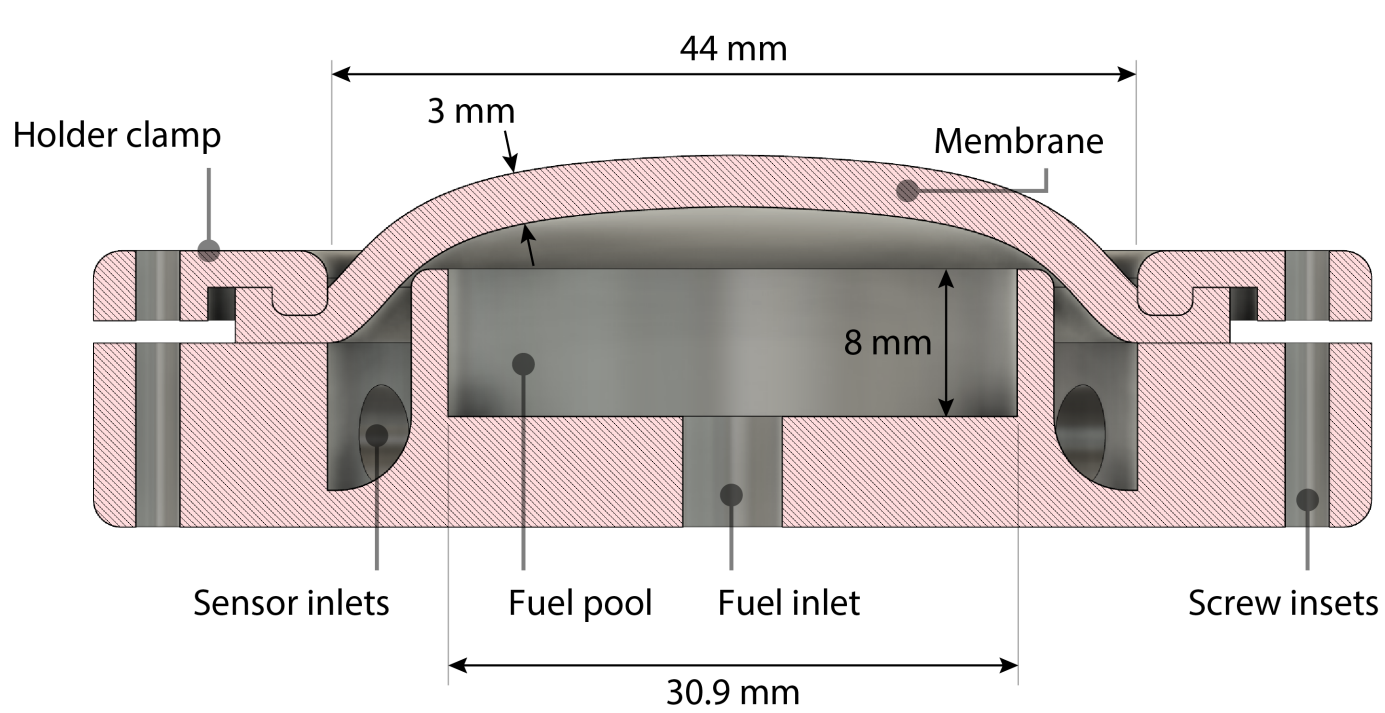


Figure S3: **Cross-section of the device.** A flat circular silicone membrane (thickness 3 mm, diameter after clamping 44 mm) is suspended over a cylindrical fuel pool (height 8 mm, internal diameter 30.9 mm) with a circular clamp. The membrane is clamped with its bottom side at a 4 mm lower height than the top of the pool, to ensure a pre-stretch. In the image, the membrane is depicted as pre-stretched, while it is flat in its resting state.

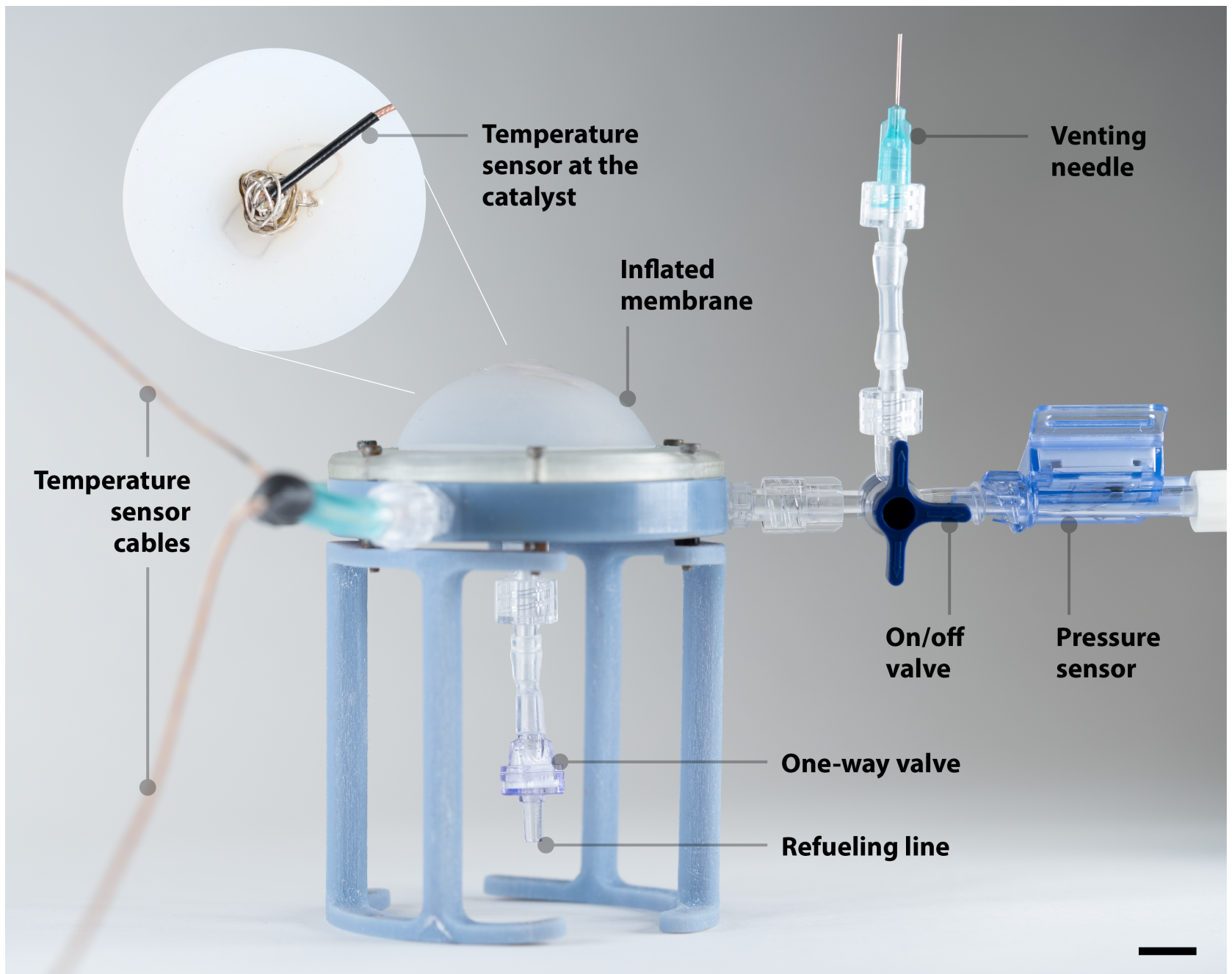


Figure S4: **The experimental setup of the active oscillator.** We connect a one-way valve on the bottom of the chamber to allow initial injection of fuel into the pool. We insert two temperature sensors: one is attached directly to the catalyst, and the second measures the temperature of the air in the chamber. A needle allows for venting the reacted gases to atmosphere. A pressure sensor measures the pressure inside the chamber. We initially keep the membrane inflated (as shown in the photograph) using an on/off valve to prevent the catalyst from activating the reaction. Scale bar is 1 cm.

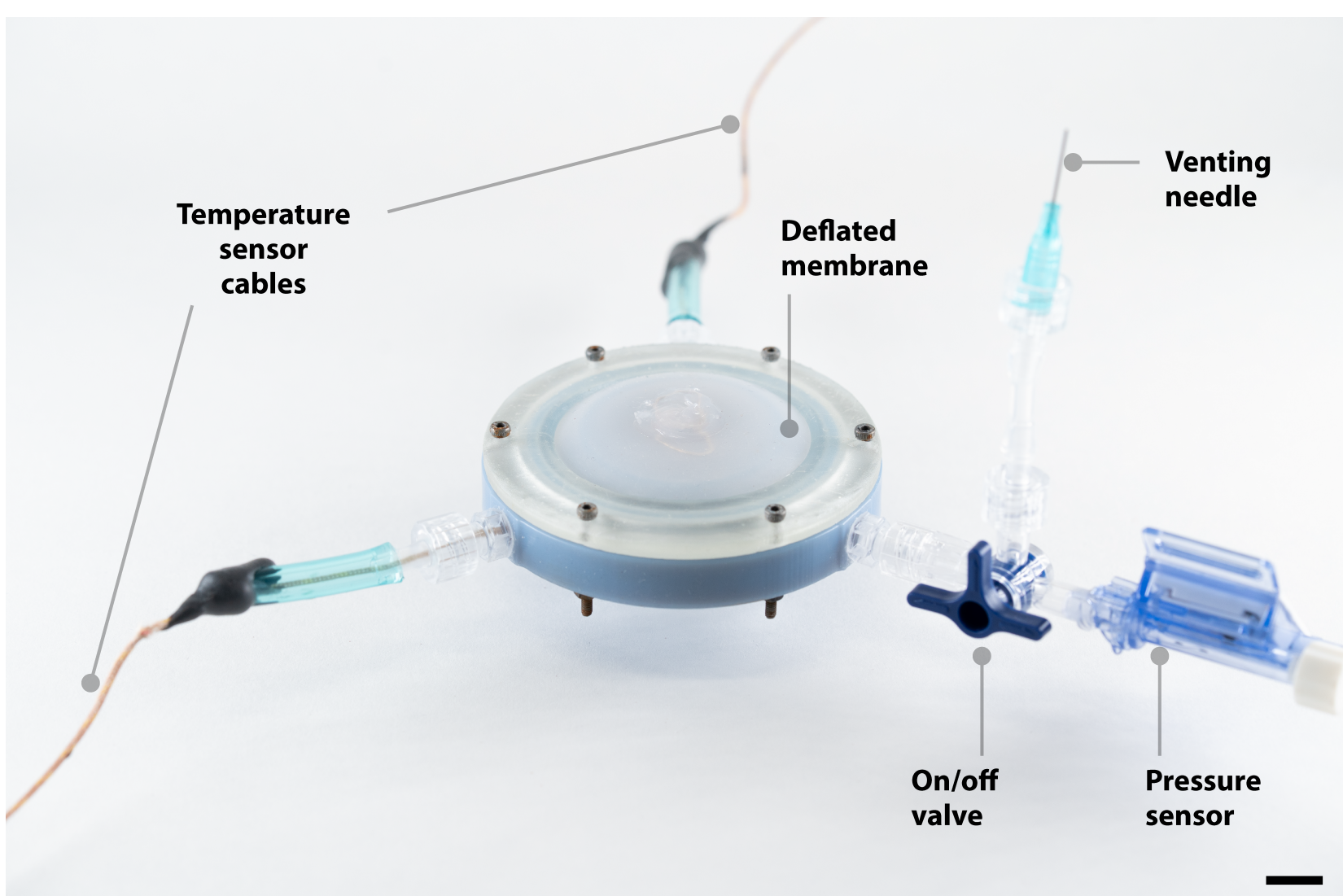


Figure S5: **Top-down view of the experimental setup of the active oscillator.** Additional view of the setup in Fig S4, without the one-way valve, highlighting the connections of the temperature sensors and pressure sensor. Scale bar is 1 cm.

## 3 Derivation of the model of the active oscillation

### Fuel mass balances

In the pool, the total mass of fuel at time $t$ is the sum of the reactable part and the inert part:

$$m_{f,p}(t) = m_{f,p}^{R}(t) + m_{f,p}^{I}(t). \tag{S1}$$

Likewise, the total fuel mass attached to the catalyst through capillary forces is the sum of the reactable and inert parts:

$$m_{f,c}(t) = m_{f,c}^{R}(t) + m_{f,c}^{I}(t). \tag{S2}$$

The reactable and inert mass of fuel in both the pool and the catalyst are state variables as in Eq. (S24), so they can evolve in time.
We define the concentration of reactable fuel in the pool $\alpha_p(t)$ as the ratio between the reactable mass and the total mass of fuel:

$$\alpha_p(t) = \frac{m_{f,p}^{R}(t)}{m_{f,p}(t)}. \tag{S3}$$

At the beginning of the experiment, the concentration of reactable fuel in the pool is $\alpha_{p_0} = 0.42$, and it will decrease over time as the reaction occurs. We measure the actual value of the initial fuel concentration by weighing with a chemical mass balance (of 0.1 mg precision) a known volume of 1 mL of fuel measured with a precision pipette, and accounting for density of water $\sim 1\,\mathrm{g/mL}$ and density of hydrogen peroxide $\sim 1.4\,\mathrm{g/mL}$. The value $\alpha_{p_0} = 0.42$ differs from the desired 0.5, possibly due to manufacturer mixing variation or gradual decomposition of fuel during two years of storage in the lab.
Likewise, we define the concentration of reactable fuel in the catalyst $\alpha_c(t)$ as the ratio between the reactable mass and the total mass (when the total mass is positive):

$$\alpha_c(t) = \begin{cases} 0 & \text{if } m_{f,c}(t) = 0, \\ \frac{m_{f,c}^{R}(t)}{m_{f,c}(t)} & \text{if } m_{f,c}(t) > 0. \end{cases} \tag{S4}$$

### Surface area of contact between catalyst and fuel

The contact between the moving catalyst and the fuel in the pool is a crucial element that affects the fuel uptake into the catalyst. During the experiment, the catalyst moves up and down, and at the same time, the height of the pool decreases as the reaction occurs: both affect the surface area of contact at time $t$.
We start by accounting for the total volume of fuel in the pool decreasing over time. The change in total volume of fuel in the pool since the start of the experiment $\Delta V_{f,p}(t)$ depends on the current concentration of fuel $\alpha_p(t)$ at time $t$, which affects the current fuel density:

$$\Delta V_{f,p}(t) = V_{f,p_0} - \frac{m_{f,p}(t)}{\rho_{H_2O_2} \cdot \alpha_p(t) + \rho_{H_2O} \cdot (1 - \alpha_p(t))}, \tag{S5}$$

with $V_{f,p_0} = 6\,\mathrm{mL}$ the initial volume of fuel in the pool, $\rho_{H_2O_2} = 1.45 \times 10^3\,\mathrm{kg/m^3}$ the density of hydrogen peroxide, and $\rho_{H_2O} = 1 \times 10^3\,\mathrm{kg/m^3}$ the density of water. This decrease in volume since the start of the experiment results in a reduced height of fuel in the pool $\Delta u_{f,p}(t)$. Knowing that the fuel pool is a cylindrical container, we get:

$$\Delta u_{f,p}(t) = \frac{\Delta V_{f,p}(t)}{r_p^2 \pi}, \tag{S6}$$

with $r_p = 15.451\,\mathrm{mm}$ the radius of the cylindrical pool.
To model the surface area of the contact between the catalyst and the fuel, we idealize the catalyst as a cylinder with radius $r_c$ and height $h_c$. At $t = t_0$, the catalyst is at zero displacement $u_0$, and is assumed to be fully dipped in the pool. When the bottom of the catalyst is above the height of the pool ($u(t) >$

$h_c - \Delta u_{f,p}(t)$), the contact area is zero. When the bottom of the catalyst is dipped underneath the pool surface, the portion of the cylinder underneath the pool surface contributes to the contact area:

$$A_s(t) = \begin{cases} 0 & \text{if } u(t) > h_c - \Delta u_{f,p}(t), \\ r_c^2\pi + 2\pi r_c \cdot (h_c - u(t) - \Delta u_{f,p}(t)) & \text{if } u(t) \leq h_c - \Delta u_{f,p}(t), \end{cases} \tag{S7}$$

in which we account for the decrease in the fuel height $\Delta u_{f,p}(t)$.

### Fuel uptake into the catalyst from the pool

When the catalyst is in contact with the fuel in the pool, the fuel transfers from the pool onto the catalyst, which we model in a simplified fashion as an effective fuel-uptake process. We do not aim to resolve a microscopic Washburn front [S1], since the catalyst geometry is irregular, moving, partially immersed, and undergoing vigorous reaction with vapor generation. Instead, we use a lumped first-order uptake law that captures the relevant system-level features: finite fuel storage in the catalyst, dependence on catalyst-fuel contact area, and a characteristic replenishment timescale. We assume that the fuel uptake is a function of just the amount of fuel present in the catalyst, and not the amount of fuel in the pool, as the mass of fuel in the pool is much larger than the mass of fuel that can be present in the catalyst. The expected rate of fuel mass entering the catalyst through fuel uptake at time $t$ is a function of the effective coefficient of fuel uptake $C_f^{\text{eff}}$ and the contact surface area $A_s(t)$, and it is zero when the mass of fuel in the catalyst reaches the maximum mass value $m_{f,c}^{\max}$ that can be held by the catalyst through capillary forces:

$$\dot{m}_{f,c}^{\text{in}}(t) = C_f^{\text{eff}} \cdot A_s(t) \cdot (m_{f,c}^{\max} - m_{f,c}(t)). \tag{S8}$$

The maximum fuel mass in the catalyst $m_{f,c}^{\max}$ is obtained from the assumed maximum fuel volume that can be held by the catalyst $V_{f,c}^{\max}$ through capillary forces and the initial fuel concentration $\alpha_{p_0}$:

$$m_{f,c}^{\max} = V_{f,c}^{\max} \cdot (\rho_{\text{H}_2\text{O}_2} \cdot \alpha_{p_0} + \rho_{\text{H}_2\text{O}} \cdot (1 - \alpha_{p_0}))\,. \tag{S9}$$

We experimentally measure an approximate value of the maximum fuel volume that the catalyst can hold $V_{f,c}^{\max}$ as follows. We first dip the catalyst in water, and then carefully undip and move the wet catalyst onto a precision scale. We compare the weight of this wet catalyst with the weight of the dry catalyst: the difference is the mass of water held by the catalyst via capillary forces. Although we recognize that the surface tension of water is lower than that of hydrogen peroxide (at 20 °C, 72.8 mN/m compared to 80.2 mN/m [S2]), we employ this method to avoid initiating the reaction while measuring the weight, which would pollute the measurement. Thus, our water-based measurement confirms experimentally that the catalyst can hold approximately 0.05 mL of liquid via capillary forces, in static conditions. We note that the fitted $V_{f,c}^{\max}$ (Table S1) is intentionally slightly below this physical holding capacity, as it represents the effective fuel reservoir during dynamic operation rather than the total liquid retained under static conditions.

Of the total expected rate of fuel mass in Eq. (S8), we distinguish between the fuel uptake of the reactable and inert parts. We model the reactable fuel flow rate from the pool entering the catalyst by accounting for the baseline capillary term, function of the pool concentration $\alpha_p(t)$, and for a factor that compensates for differences in concentration between the fuel present in the catalyst and in the pool:

$$\dot{m}_{f,c}^{R,\text{in}}(t) = \dot{m}_{f,c}^{\text{in}}(t) \cdot \alpha_p(t) + C_f^{\text{eff}} \cdot A_s(t) \cdot m_{f,c}^{\max} \cdot (\alpha_p(t) - \alpha_c(t)). \tag{S10}$$

To reach the expected total fuel flow rate, we define the inert fuel flow rate as the difference between the expected (Eq. (S8)) and the reactable (Eq. (S10)) rates:

$$\dot{m}_{f,c}^{I,\text{in}}(t) = \dot{m}_{f,c}^{\text{in}}(t) - \dot{m}_{f,c}^{R,\text{in}}(t). \tag{S11}$$

### Gas produced from the reaction

We assume that the mass of hydrogen peroxide on the catalyst $m_{f,c}^R$ decomposes into only gas mass $m_g^{\text{gen}}$ (water vapor and oxygen). The hydrogen peroxide decomposition is a first-order catalytic reaction [S3]:

$$\dot{m}_g^{\text{gen}}(t) = k_r(T_c(t)) \cdot m_{f,c}^R(t). \tag{S12}$$

The reaction is temperature-dependent, where the rate $k_r$ is an exponential function of temperature $T_c$ as per the Arrhenius equation [S4]. For simplicity, we assume a linear relationship between the reaction rate and the catalyst temperature $T_c$:

$$k_r(T_c(t)) = k_{r_0} + C_r \cdot (T_c(t) - T_0), \tag{S13}$$

with $T_0$ the ambient temperature, $k_{r_0}$ the baseline reaction rate at ambient temperature, and $C_r$ the reaction constant.

### Pressure and gas leakage

While the reaction occurs and gases are being generated inside the chamber, at the same time, gas leaks through the venting needle, inducing a gas mass loss:

$$\dot{m}_g^{\text{leak}}(t) = \frac{p(t)}{R_l}, \tag{S14}$$

with $p(t)$ the pressure inside the chamber and $R_l$ a constant that represents the fluidic resistance of the venting needle. The pressure inside the chamber at time $t$ is a function of the total mass of gas $m_g(t)$, under the assumption of ideal gas:

$$p(t) = \frac{m_g(t)}{M_g} \cdot \frac{R \cdot T_0}{V_{\text{ch}}}, \tag{S15}$$

where $M_g$ is the molar mass of the reacted gas, $R = 8.314\,\text{J/(K mol)}$ is the gas constant, and $V_{\text{ch}} = 24 \times 10^{-6}\,\text{m}^3$ is the volume of the chamber, which we assume to be constant for simplicity, despite it actually varying in the order of magnitude of 1 mL in experiments, as the membrane deforms when pressurized. We calculate $M_g = 0.022\,667\,\text{kg/mol}$ as the molar mass of water vapor and oxygen mixture at a ratio of 2:1 moles, based on the simplifying assumption that the product of the reaction is only gas (without liquid water), and based on the 2:1 molar ratio of the decomposition in Eq. (1). The gas mass balance (Fig. 4b) becomes:

$$\dot{m}_g(t) = \dot{m}_g^{\text{gen}}(t) - \dot{m}_g^{\text{leak}}(t). \tag{S16}$$

### Thermal balance

The catalytic reaction generates heat as a function of the heat of decomposition of the exothermic reaction $H_{\text{dec}} = 2884.5\,\text{kJ/kg}$ [S5] and the rate of generation of gas:

$$\dot{Q}^{\text{gen}}(t) = H_{\text{dec}} \cdot \dot{m}_g^{\text{gen}}(t). \tag{S17}$$

Simultaneously, the catalyst dissipates heat as a function of its temperature:

$$\dot{Q}^{\text{loss}}(t) = C_{\text{loss}} \cdot (T_c(t) - T_0), \tag{S18}$$

with $C_{\text{loss}}$ the heat loss coefficient.
The effective heat capacity of the catalyst $M_C(t)$ sees contributions from the silver mass $m_c$ and the residual mass of water present on the catalyst $m_{f,c}^I(t)$:

$$M_C(t) = m_c \cdot c_{p,s} + m_{f,c}^I(t) \cdot c_{p,w}, \tag{S19}$$

with $c_{p,s} = 235\,\text{J/(kg}\,^\circ\text{C)}$ the specific heat capacity of silver and $c_{p,w} = 4200\,\text{J/(kg}\,^\circ\text{C)}$ the specific heat capacity of water. The temperature dynamics (Fig. 4c) follow:

$$\dot{T}_c(t) = \frac{\dot{Q}^{\text{gen}}(t) - \dot{Q}^{\text{loss}}(t)}{M_C(t)}. \tag{S20}$$

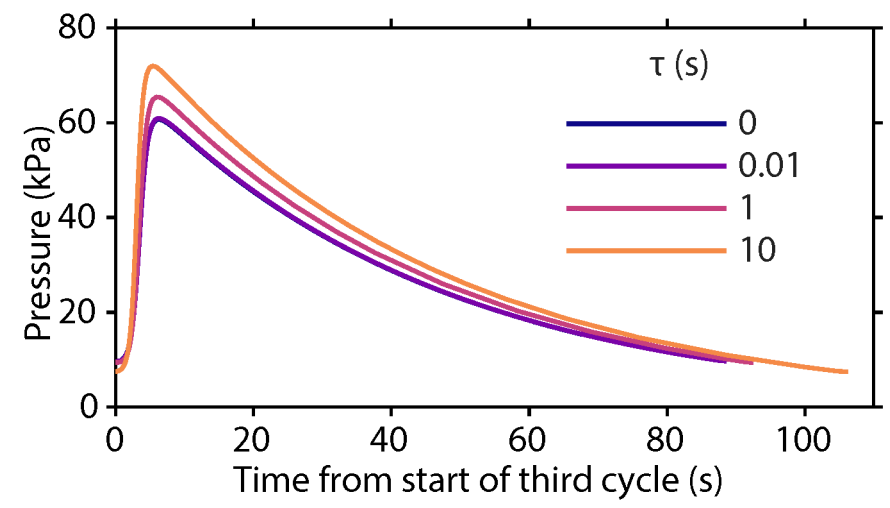


Figure S6: **Effect of strong viscoelasticity over one oscillation cycle.** Evolution of pressure over the third oscillation cycle, for various damping coefficients $C_d = \tau \cdot k_s$, with $k_s = 2.17 \times 10^3$ N/m and $\tau$ ranging from 0 s to 10 s. The corresponding damping coefficients $C_d$ are 0, $2.2 \cdot 10^1$, $2.2 \cdot 10^3$, and $2.2 \cdot 10^4$ N s/m.

## Mechanics of the membrane

We idealize the membrane as a vertical linear spring connected to the catalyst, with a damper in parallel to account for dissipative forces such as friction and other viscous effects (Fig. 4a, blue). We model the pressurization of the membrane as a force applied to the spring, which is proportional to the internal pressure in the chamber $p(t)$ and the surface area of the membrane $A_m$:

$$F_p(t) = A_m \cdot p(t). \tag{S21}$$

The dynamics of the catalyst attached to the spring follow Newton's second law:

$$m_c \cdot \ddot{u}(t) = -k_s \cdot u(t) - C_d \cdot \dot{u}(t) + F_p(t), \tag{S22}$$

with $m_c$ the mass of the catalyst, $k_s$ the spring stiffness, and $C_d$ the damping coefficient. We assign $m_c =$ 1.2 g as measured. We estimate the spring stiffness as $k_s = (p^* \cdot A^*)/u^* \sim 2.2 \times 10^3$ N/m (Table S1), by measuring experimentally that the membrane apex displaces upwards by $u^* = 7$ mm under an internal pressure $p^* = 10$ kPa applied over an approximate membrane area of $A^* = \pi(D^*/2)^2 = 1.5 \times 10^3\,\text{mm}^2$, where $D^* = 44$ mm is the membrane diameter.

We assign a damping coefficient $C_d = 0$ N s/m given the following observations. We note that the critical damping value of such a mass-spring system is $C_{cr} = 2\sqrt{m_c \cdot k_s}$ which equals 3.2 N s/m when substituting our values. When varying $C_d$ from 0 to $3 \cdot C_{cr}$—effectively spanning both underdamped and overdamped cases—we do not notice an appreciable change in the active oscillation response. This is because the mechanical timescale (undamped natural period) is in the order of $2\pi/\sqrt{k_s/m_c} \sim 5$ ms, while the overall active oscillation period of the whole elasto-chemical oscillator is in the order of minutes. The elasto-chemical cycle is therefore several orders of magnitude slower than the free mechanical response of the membrane. This means that other dissipative factors—likely, the venting resistance that slowly vents gases to the atmosphere—are dominating the viscous response of the overall coupled system.

We then further probe strongly viscoelastic mechanical responses, with damping coefficients orders of magnitude above the critical damping factor. For a strongly overdamped system, the relevant slow mechanical relaxation time is approximately $\tau \approx C_d/k_s$. To allow damping to have a visible effect over a cycle lasting tens of seconds, we probe $\tau$ so that it is comparable to the entire elasto-chemical timescale, which is in the order of tens of seconds. We therefore sweep $\tau$ from 0 s to 10 s, and run individual simulations with the corresponding $C_d = \tau \cdot k_s$, and all other parameters unchanged. In Fig. S6, we report the third cycle of each simulation. From Fig. S6, we note that strong viscoelastic responses would quantitatively affect the active oscillation only marginally, with a relatively small increase in period and amplitude. However, no considerable qualitative changes are appreciable, even in this case of strong simulated viscoelasticity.

We conclude that, because the material viscous dissipation plays a marginal role relative to the overall oscillation timescale, we can safely neglect viscoelasticity without losing key factors underlying the active oscillation behavior. Hence, throughout the manuscript, we assume $C_d = 0$ N s/m, meaning that we approximate our membranes as ideally elastic.

### System of equations

Following the above relationships, we write the system of equations:

$$\begin{cases} \dot{m}_{f,p}^{R}(t) = -\dot{m}_{f,c}^{R,\text{in}}(t) \\ \dot{m}_{f,p}^{I}(t) = -\dot{m}_{f,c}^{I,\text{in}}(t) \\ \dot{m}_{f,c}^{R}(t) = \dot{m}_{f,c}^{R,\text{in}}(t) - \dot{m}_{g}^{\text{gen}} \\ \dot{m}_{f,c}^{I}(t) = \dot{m}_{f,c}^{I,\text{in}}(t) \\ \dot{m}_{g}(t) = \dot{m}_{g}^{\text{gen}}(t) - \dot{m}_{g}^{\text{leak}}(t) \\ \dot{T}_{c}(t) = \left[\dot{Q}^{\text{gen}}(t) - \dot{Q}^{\text{loss}}(t)\right]/M_{C}(t) \\ \dot{u}(t) = \dot{u}(t) \\ \ddot{u}(t) = \left[-k_{s}\cdot u(t) - C_{d}\cdot\dot{u}(t) + F_{p}(t)\right]/m_{c} \end{cases} . \tag{S23}$$

### The complete set of ODEs

Following the definitions of the state variables in Figure 4, we define the state vector as:

$$\begin{aligned} \mathbf{s}(t) &= [\ s_1(t)\ ,\ s_2(t)\ ,\ s_3(t)\ ,\ s_4(t)\ ,\ s_5(t)\ ,\ s_6(t)\ ,\ s_7(t)\ ,\ s_8(t)\ ] \\ &= [\ m_{f,p}^{R}(t)\ ,\ m_{f,p}^{I}(t)\ ,\ m_{f,c}^{R}(t)\ ,\ m_{f,c}^{I}(t)\ ,\ m_{g}(t)\ ,\ T_{c}(t)\ ,\ u(t)\ ,\ \dot{u}(t)\ ]. \end{aligned} \tag{S24}$$

Starting from the set of Equations (S23), we plug in the relationships in Equations (S10), (S11), (S12), (S14), (S17), (S18), (S19), (S21), and then the rest of the needed equations as described in Section 4 in the main text, obtaining a set of 8 ODEs:

$$\begin{cases} \dot{s}_1 = -C_f^{\text{eff}}\cdot A_s(s_1,s_2,s_7)\cdot S_{\text{react}}^{\text{in}}(s_1,s_2,s_3,s_4) \\ \dot{s}_2 = C_f^{\text{eff}}\cdot A_s(s_1,s_2,s_7)\cdot S_{\text{inert}}^{\text{in}}(s_1,s_2,s_3,s_4) \\ \dot{s}_3 = C_f^{\text{eff}}\cdot A_s(s_1,s_2,s_7)\cdot S_{\text{react}}^{\text{in}}(s_1,s_2,s_3,s_4) - s_3\cdot\left[k_{r_0} + C_r\cdot(s_6 - T_0)\right] \\ \dot{s}_4 = -C_f^{\text{eff}}\cdot A_s(s_1,s_2,s_7)\cdot S_{\text{inert}}^{\text{in}}(s_1,s_2,s_3,s_4) \\ \dot{s}_5 = s_3\cdot\left[k_{r_0} + C_r\cdot(s_6 - T_0)\right] - \dfrac{R\cdot T_0}{R_l\cdot M_g\cdot V_{\text{ch}}}\cdot s_5 \\ \dot{s}_6 = \dfrac{H_{\text{dec}}\cdot s_3\cdot\left[k_{r_0} + C_r\cdot(s_6 - T_0)\right] - C_{\text{loss}}\cdot(s_6 - T_0)}{m_c\cdot c_{p,s} + c_{p,w}\cdot s_4} \\ \dot{s}_7 = s_8 \\ \dot{s}_8 = \dfrac{1}{m_c}\cdot\left(-k_s\cdot s_7 - C_d\cdot s_8 + \dfrac{A_m\cdot R\cdot T_0}{M_g\cdot V_{\text{ch}}}\cdot s_5\right) \end{cases} , \tag{S25}$$

with

$$S_{\text{react}}^{\text{in}}(s_1,s_2,s_3,s_4) = m_{f,c}^{\max}\cdot\left(\frac{2s_1}{s_1+s_2} - \frac{s_3}{s_3+s_4}\right) - \frac{s_1\cdot(s_3+s_4)}{s_1+s_2}, \tag{S26}$$

and

$$S_{\text{inert}}^{\text{in}}(s_1,s_2,s_3,s_4) = m_{f,c}^{\max}\cdot\left(\frac{2s_1}{s_1+s_2} - \frac{s_3}{s_3+s_4} - 1\right) - \frac{s_1\cdot(s_3+s_4)}{s_1+s_2} + s_3 + s_4, \tag{S27}$$

and $m_{f,c}^{\max}$ as defined in Eq. (S9), and

$$A_s(s_1,s_2,s_7) = \begin{cases} 0 & \text{if } s_7 > h_c - \Delta u_{f,p}(t), \\ r_c^2\pi + 2\pi r_c\cdot(h_c - s_7 - \Delta u_{f,p}(t)) & \text{if } s_7 \le h_c - \Delta u_{f,p}(t), \end{cases} \tag{S28}$$

where

$$\Delta u_{f,p}(t) = \frac{1}{r_p^2 \pi} \cdot \left( V_{f,p_0} - \frac{s_1 + s_2}{\rho_{\mathrm{H_2O_2}} \cdot \frac{s_1}{s_1+s_2} + \rho_{\mathrm{H_2O}} \cdot \frac{s_2}{s_1+s_2}} \right). \tag{S29}$$

In Eq. (S25), (S26), (S27), (S28), and (S29), only the state variables $s_1$, $s_2$, $s_3$, $s_4$, $s_5$, $s_6$, $s_7$, and $s_8$ depend on time, while the others are fixed parameters summarized in Table S1.
Before numerically integrating the ODE in Eq. (S25) with the MATLAB solver `ode23s`, we initialize the state variables by considering: *i)* a known initial amount of fuel in the pool with volume $V_{f,p_0}$, *ii)* a known initial concentration of fuel in the pool $\alpha_{p_0}$, *iii)* no fuel in the catalyst ($m^R_{f,c}(0) = m^I_{f,c}(0) = 0$), *iv)* catalyst at room temperature ($T_c(0) = T_0$), and *v)* spring at rest ($u(0) = \dot{u}(0) = 0$). Hence, the state variables at time $t = 0$ are:

$$\begin{aligned}
s_1(0) &= V_{f,p_0} \cdot \alpha_{p_0} = V_{f,p_0} \cdot (\rho_{\mathrm{H_2O_2}} \cdot \alpha_{p_0} + \rho_{\mathrm{H_2O}} \cdot (1-\alpha_{p_0})) \cdot \alpha_{p_0} \\
s_2(0) &= V_{f,p_0} \cdot (1-\alpha_{p_0}) = V_{f,p_0} \cdot (\rho_{\mathrm{H_2O_2}} \cdot \alpha_{p_0} + \rho_{\mathrm{H_2O}} \cdot (1-\alpha_{p_0})) \cdot (1-\alpha_{p_0}) \\
s_3(0) &= 0 \\
s_4(0) &= 0 \\
s_5(0) &= 0 \\
s_6(0) &= T_0 \\
s_7(0) &= 0 \\
s_8(0) &= 0
\end{aligned} \quad . \tag{S30}$$

**A phase diagram of the active oscillation over positive and negative feedback**

To draw a phase diagram of the active oscillation, we chose two model parameters that are relevant when discussing positive and negative feedback. Looking at the schematic in Fig. 5f, we note that *i)* a stronger leakage of gas mass $m_g^{\mathrm{leak}}$ promotes negative feedback by accelerating the membrane return to the pool surface, and *ii)* a larger catalyst fuel mass $m_{f,c}$ promotes positive feedback by allowing more gases to be generated, inducing higher internal pressure. Hence, we examine the two model parameters that most strongly affect these quantities: a smaller venting resistance $R_l$ promotes negative feedback, and a larger maximum catalyst fuel volume $V_{f,c}^{\mathrm{max}}$ promotes positive feedback.
In Fig. S7a, we report a phase diagram of the active oscillation as the oscillation frequency function of decreasing $R_l$ and increasing $V_{f,c}^{\mathrm{max}}$, both spanning 4 orders of magnitude. We notice how too strong or too weak feedback (either positive or negative) suppresses the oscillation. This effect is particularly evident in the cases of strong positive feedback ($V_{f,c}^{\mathrm{max}} \gtrapprox 0.3\,\mathrm{mL}$): in this region, large domains of negative feedback (weak, $R_l \gtrapprox 1 \times 10^{12}\,\mathrm{Pa\,s/kg}$ and strong, $R_l \lessapprox 1 \times 10^{11}\,\mathrm{Pa\,s/kg}$) suppress the oscillation. This behavior is evident in Fig. S7b-d, where we report the pressure evolution over 45 minutes for three cases of $R_l$ given a large $V_{f,c}^{\mathrm{max}}$. For weak negative feedback (Fig. S7b), the sole initial oscillation slowly decays, because the return of the membrane is inhibited by the high venting resistance. When the negative feedback effects balance the positive feedback effects, the active oscillation is sustained (Fig. S7c). For strong negative feedback (Fig. S7d), the initial oscillation decays rapidly, and the catalyst remains attracted around the pool surface, because the reacted gases rapidly vent to atmosphere.

# 4 Supplementary figures and tables

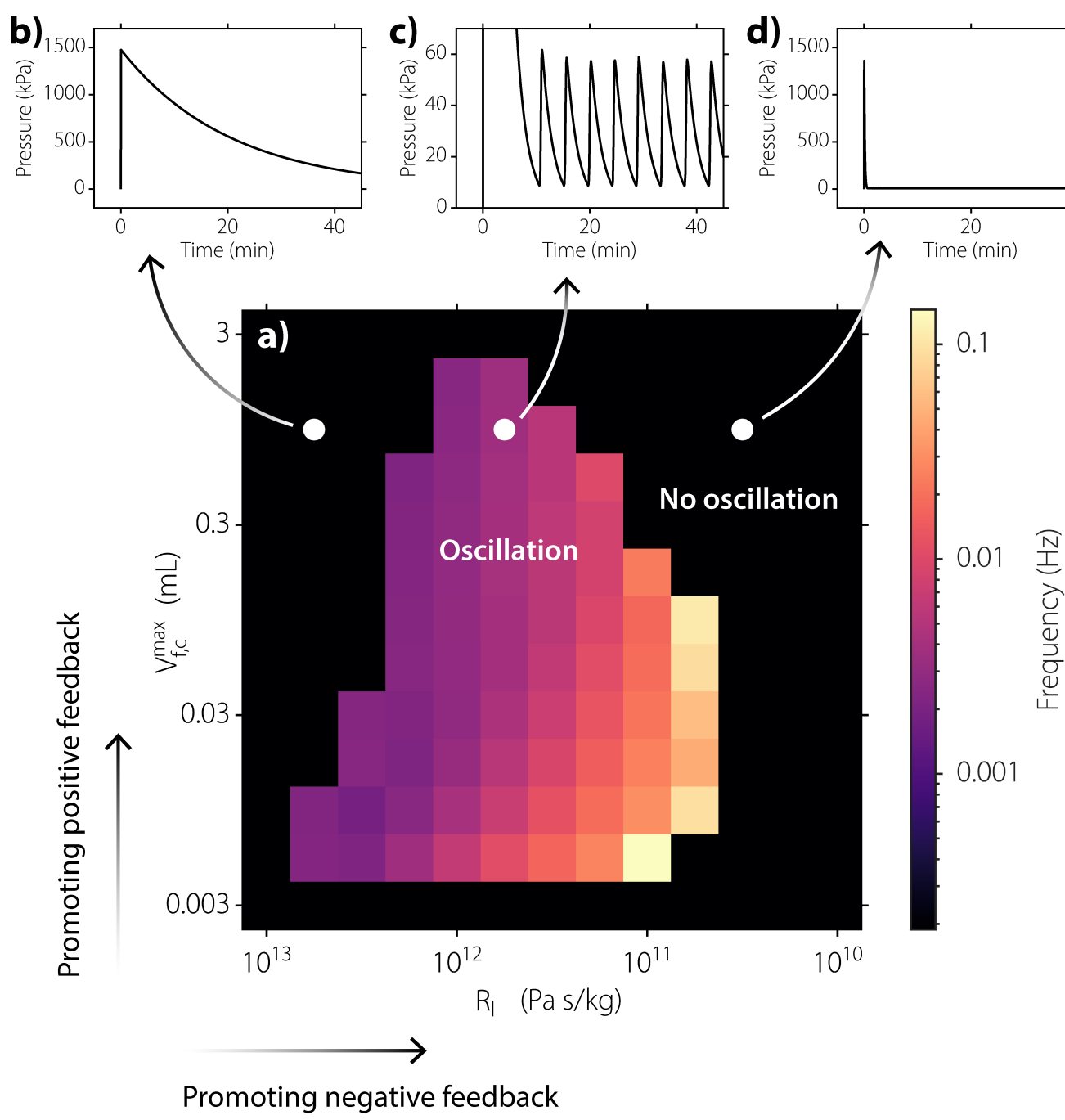


Figure S7: **Phase diagram of the active oscillation over positive and negative feedback.** (**a**) Oscillation frequency as a function of decreasing venting resistance $R_l$ (which promotes negative feedback) and of increasing maximum catalyst fuel volume $V_{f,c}^{\max}$ (which promotes positive feedback). Each datapoint in the phase diagram is the average oscillation frequency of a simulation lasting 150 simulated minutes. (**b**) Pressure evolution for $V_{f,c}^{\max} = 0.95\,\text{mL}$ and $R_l = 5.6 \times 10^{12}\,\text{Pa s/kg}$. (**c**) Pressure evolution for $V_{f,c}^{\max} = 0.95\,\text{mL}$ and $R_l = 5.6 \times 10^{11}\,\text{Pa s/kg}$. (**d**) Pressure evolution for $V_{f,c}^{\max} = 0.95\,\text{mL}$ and $R_l = 3.2 \times 10^{10}\,\text{Pa s/kg}$.

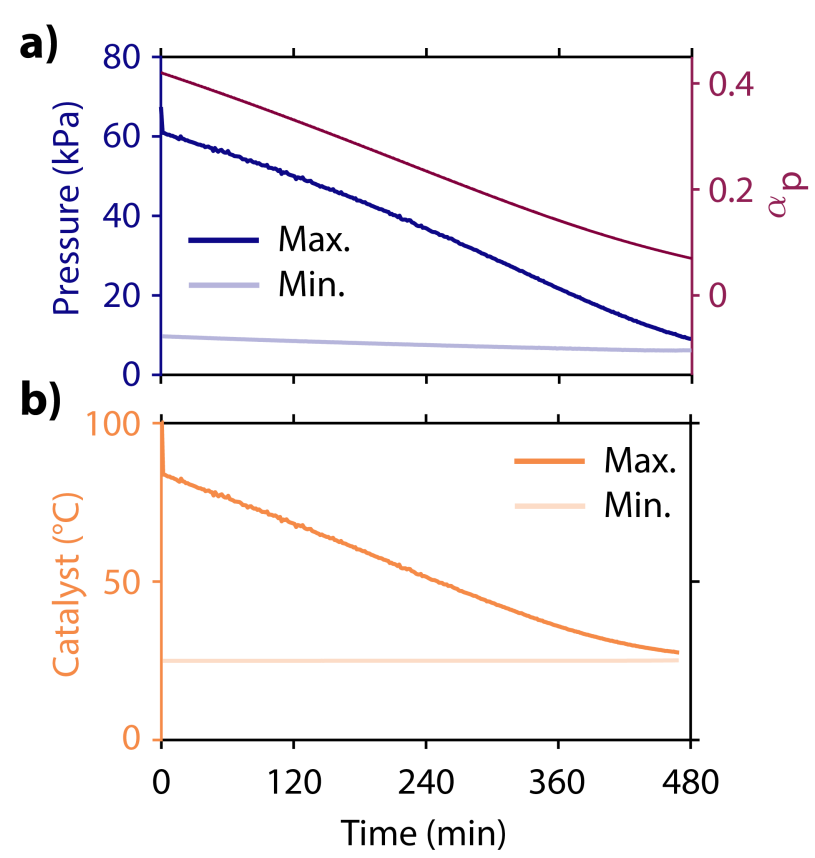


Figure S8: **Evolution over 8 hours of the simulation.** (**a**) The maximum and minimum pressure values (blue) and fuel concentration in the pool (dark red) decrease over 8 simulated hours. (**b**) The maximum catalyst temperature decreases over 8 hours, while the minimum catalyst temperature stays around ambient temperature. The parameters used in these simulations are listed in Table S1.

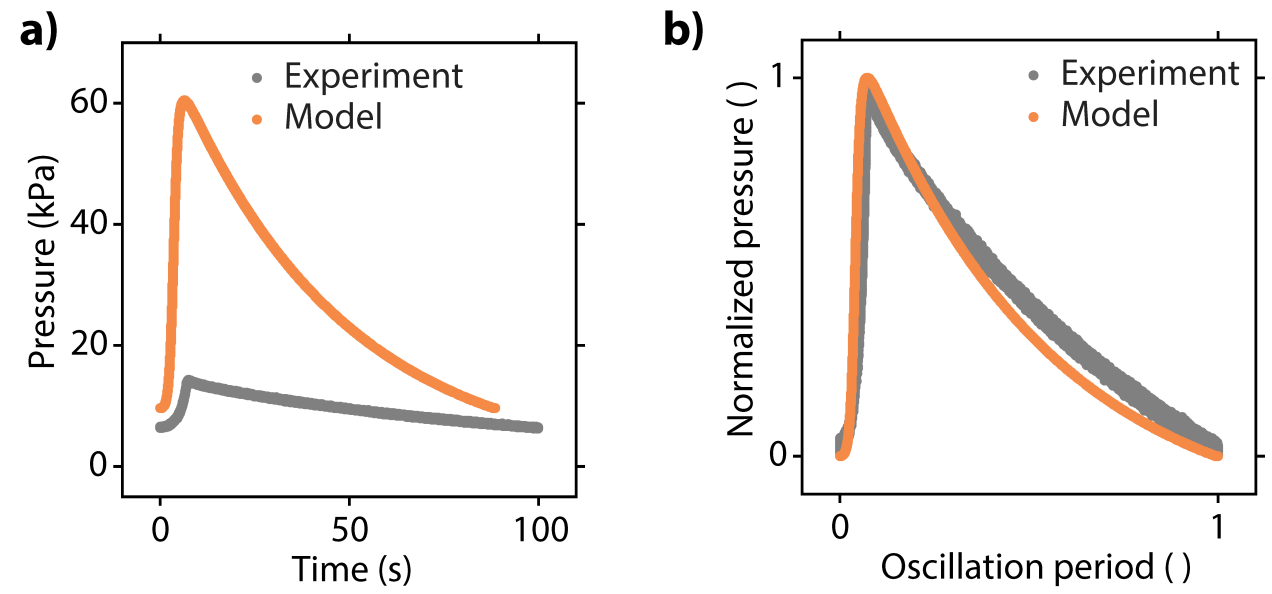


Figure S9: **Comparison between experimental and simulated pressure, for one active oscillation cycle.** (**a**) Experimental pressure evolution during an oscillation cycle at quasi-steady-state conditions (cycle at 60 minutes of the experiment in Figure 3) (grey), compared with the fourth cycle of the simulation in Figure 5 (orange). Note that the model does not capture the initial transient, but only the steady state, hence our selection of one of the initial simulated cycles for the comparison. (**b**) Same data as in **a**, normalized over pressure amplitude and oscillation period.

Table S1: **Parameters of the simulations in Figures 5, 6, 7.** We refer to each parameter as 'known' when it is a scientifically established value, 'measured' when we experimentally measure it in the lab for the test conditions of the experiments in Figure 2 and 3, or 'fitted' when we assign it to qualitatively fit the simulation to the experiment in Figure 2, without aiming to fit the long-term evolution of the oscillations of Figure 3.

| Param. | Value | Unit | Description | Motivation |
|---|---|---|---|---|
| $T_0$ | 298.15 | K | Room temperature | Measured |
| $R$ | 8.314 | J/(K mol) | Gas constant | Known |
| $M_g$ | 0.022667 | kg/mol | Molar mass of gas | Known |
| $\rho_{H_2O}$ | $1 \times 10^3$ | kg/m$^3$ | Density of water | Known |
| $\rho_{H_2O_2}$ | $1.45 \times 10^3$ | kg/m$^3$ | Density of $H_2O_2$ | Known |
| $c_{p,s}$ | 235 | J/(kg °C) | Silver specific heat capacity | Known |
| $c_{p,w}$ | 4200 | J/(kg °C) | Water specific heat capacity | Known |
| $H_{\mathrm{dec}}$ | $2.8845 \times 10^6$ | J/kg | $H_2O_2$ decomposition heat | Ref. [S5] |
| $C_{\mathrm{loss}}$ | 0.1 | J/(K s) | Heat loss coefficient | Fitted |
| $\alpha_{p_0}$ | 0.42 | – | Initial fuel concentration | Measured |
| $V_{\mathrm{ch}}$ | $24 \times 10^{-6}$ | m$^3$ | Initial chamber volume | Measured |
| $V_{f,p_0}$ | $6 \times 10^{-6}$ | m$^3$ | Initial fuel volume in pool | Measured |
| $r_p$ | $15.451 \times 10^{-3}$ | m | Radius of pool | Measured |
| $r_c$ | $4.5 \times 10^{-3}$ | m | Radius of catalyst | Measured |
| $h_c$ | $7 \times 10^{-3}$ | m | Height of catalyst | Measured |
| $m_c$ | $1.2 \times 10^{-3}$ | kg | Mass of catalyst | Measured |
| $V_{f,c}^{\max}$ | $0.03 \times 10^{-6}$ | m$^3$ | Catalyst maximum fuel volume | Fitted |
| $C_f^{\mathrm{eff}}$ | $1 \times 10^4$ | 1/(m$^2$ s) | Effective fuel-uptake coefficient | Fitted |
| $C_r$ | 0.02 | 1/(K s) | Reaction constant | Fitted |
| $k_{r_0}$ | $1 \times 10^{-3}$ | 1/s | Reaction rate at $T_0$ | Fitted |
| $R_l$ | $0.2 \times 10^{12}$ | Pa s/kg | Venting resistance | Fitted |
| $k_s$ | $2.17 \times 10^3$ | N/m | Spring stiffness | Measured |
| $C_d$ | 0 | N s/m | Damping coefficient | Fitted |

# References


[S1] T. L. Staples, D. G. Shaffer, *Colloids and Surfaces A: Physicochemical and Engineering Aspects* **2002**, *204*, 1-3 239.

[S2] M. Jalaal, B. ten Hagen, H. le The, C. Diddens, D. Lohse, A. Marin, *Physical Review Fluids* **2022**, *7*, 11 110514.

[S3] L. Pirault-Roy, C. Kappenstein, M. Guerin, R. Eloirdi, N. Pillet, *Journal of Propulsion and Power* **2002**, *18*, 6 1235.

[S4] J. Corpening, S. Heister, W. Anderson, B. Austin, In *40th AIAA/ASME/SAE/ASEE Joint Propulsion Conference and Exhibit*. **2004** 3373.

[S5] J. Mok, W. Helms, J. Sisco, W. Anderson, *Journal of propulsion and power* **2005**, *21*, 5 942.
